# Innovative Physical and Chemical Strategies for the Modification and Development of Polymeric Microfiltration Membranes—A Review

**DOI:** 10.3390/polym18030311

**Published:** 2026-01-23

**Authors:** Mohammad Ebrahimi

**Affiliations:** Institute CARMeN UMR 6064, ENSICAEN, University of Caen Normandy, 6 Bd. du Maréchal Juin, 14050 Caen, France; mohammad.ebrahimi@ensicaen.fr

**Keywords:** microfiltration, modification methods, polymer blending, nanomaterials incorporation, surface coating, surface functionalization, chemical crosslinking

## Abstract

Polymeric microfiltration membranes are among the most utilized pressure-driven membranes due to their excellent permeation flux, moderate removal efficiency, low operating pressure, low cost, as well as their potential for reusability and cleanability. Therefore, these membranes are used in different crucial sectors, including the water and wastewater, dairy, beverage, and pharmaceutical industries. However, well-known polymeric microfiltration membranes suffer from their poor hydrophilic properties, causing fouling phenomenon. A reduction in permeate flux, a shortened operational lifespan, and increased energy consumption are the primary negative consequences of membrane fouling. Over the years, a broad spectrum of studies has been performed to modify polymeric microfiltration membranes to improve their hydrophilic, transport, and antifouling characteristics. Despite extensive research, this issue remains a subject of ongoing discussion and scrutiny within the scientific community. This review article provides promising information about different physical and chemical modification methods—such as polymer blending, the incorporation of nanomaterials, surface coating, chemical crosslinking, in situ nanoparticle immobilization, and chemical surface functionalization—for polymeric microfiltration membranes. The physical and chemical modification methods are comparatively evaluated, highlighting their positive and negative aspects, supported by findings from recent investigations. Moreover, promising ideas and future-oriented techniques were proposed to obtain polymeric microfiltration membranes containing superior efficiency, extended service life, and mechanical strength.

## 1. Introduction

Nowadays, water scarcity and water contamination are two serious threats to human life [1,2,3,4]. Over the centuries, humans have exploited and polluted most water sources, making it increasingly difficult to find clean water today [5,6]. A significant portion of the world’s freshwater resources is consumed by agriculture and industry [7,8]. Furthermore, over the past several decades, wastewater from industrial, agricultural, and urban sources—contaminated with toxic and hazardous compounds—have frequently been insufficiently treated [9,10,11,12]. Such conditions adversely reduce water quality, disrupt aquatic ecosystems, and pose serious health risks (i.e., cancer, kidney failure, nervous system damage, cholera, typhoid fever, and dysentery) [6,13,14,15]. Consequently, there is an urgent need to develop effective methods for treating and purifying water [16,17,18,19]. Among the different techniques for water treatment, membrane technology is widely recognized for its effectiveness and versatility [20,21,22].

Today, the widespread use of pressure-driven membrane separation processes—such as microfiltration (MF), ultrafiltration (UF), nanofiltration (NF), and reverse osmosis (RO) membranes—has increased for water treatment and purification owing to their exceptional separation and transport features [23,24,25,26,27]. In particular, MF membranes have been classically utilized in different industries, including the water and wastewater, dairy, beverage, and pharmaceutical industries to remove suspended solids, bacteria, and colloidal particles from various liquid streams [26,28,29,30]. MF membranes are usually well-known membranes owing to their high permeate flux and moderate selectivity due to the fact that these membranes have a larger pore size (between 0.1 and 10 μm) in comparison with UF and NF membranes [31,32,33]. Moreover, cost-effectiveness and reduced energy consumption are two notable benefits of MF membranes compared with UF, NF, and RO membranes [34,35,36].

**Figure 1:**
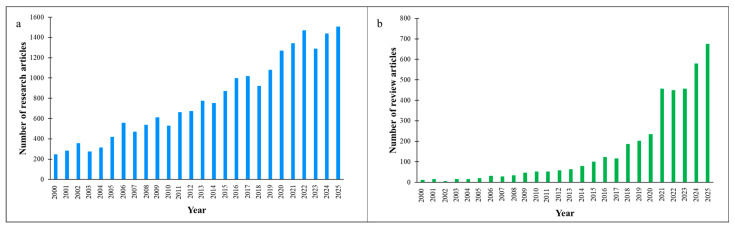
Published research (**a**) and review (**b**) articles devoted to the advancement and modification of MF membranes from 2000 to 2025 (source: Scopus database, January 2026).

**Figure 2:**
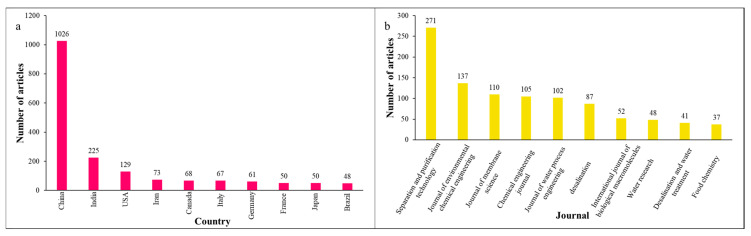
Distribution of publications on MF membranes in 2025: (**a**) top ten countries by number of published articles and (**b**) the ten journals with the largest number of publications in this field. (source: Scopus database, January 2026).

**Figure 3:**
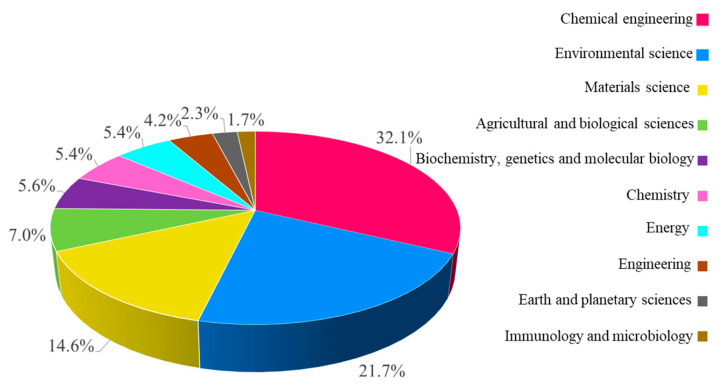
Proportional distribution of published items (research articles, review articles, and book chapters) in the top ten subject areas related to MF membranes (from 2015 to 2025) (source: Scopus database, January 2026).

Based on the used material for membrane fabrication, MF membranes are generally categorized in two classifications, polymeric- and ceramic-based membranes, which have their own unique properties [37,38,39,40]. Polyvinylidene fluoride (PVDF), polyamide-6 (PA6), polysulfone (PS), polypropylene (PP), and cellulose acetate are the most commonly used polymers for MF preparation offering great film-forming properties, flexibility, scalability potential, and cost-effectiveness [41,42,43,44,45]. Ceramic membranes are usually manufactured from alumina (Al_2_O_3_), zirconia (ZrO_2_), and/or silica (SiO_2_) [46,47,48,49,50]. This group of MF membranes shows better thermal and chemical stability as well as a longer operational life compared with polymeric MF membranes [51,52,53,54,55]. However, polymer-based MF membranes are the most commonly employed MF membranes [56,57,58,59,60]. Figure 1 illustrates the number of research and review articles regarding the advancement and modification of MF membranes from 2000 to 2025. Upward trends are observed in the number of publications over this period, revealing growing interest in this research field. Moreover, Figure 2 demonstrates the global distribution of MF membrane research output in 2025, which illustrates (a) top ten countries by number of published articles and (b) the ten journals with the largest number of publications in this field. These findings reveal that this research topic has been geographically expanding in the world, especially in high-tech countries. Moreover, it can be noticed that a substantial number of articles were published in prestigious journals (with high impact factors), indicating the importance of the MF membrane research topic within the scientific community. Furthermore, Figure 3 illustrates the proportional distribution of published items (research articles, review articles, and book chapters) in the top ten subject areas related to MF membranes (from 2015 to 2025). This figure indicates the multidisciplinary nature of this research topic in different fields, such as chemical engineering, environmental science, materials science, chemistry, energy, agricultural and biological sciences, etc.

Notwithstanding the mentioned benefits, polymeric MF membranes have suffered from fouling phenomenon mainly owing to the weak hydrophilic properties of employed polymers (i.e., PVDF, PP, PS, and PES) [61,62,63,64,65,66,67]. Membrane fouling occurs when the surface of the membrane is attractive (i.e., hydrophobic) to contaminants [68,69,70,71,72]. Therefore, a cake layer is formed on the surface of the membrane which reduces permeate flux, increases energy consumption, and decreases service life [73,74,75,76,77]. In order to maintain the separation performance of MF membranes, cleaning strategies can be used which are classified into physical (i.e., backwashing, static immersion, mechanical scraping, and sonication) and chemical (i.e., acid and alkaline cleanings) strategies [32,78,79,80,81]. However, disrupting the separation process and increasing the operational costs are the main limitations of cleaning strategies [82,83,84,85]. To address these issues, a number of studies have been devoted to develop and modify polymeric MF membranes with optimal characteristics, such as high permeate flux; excellent hydrophilicity; great mechanical, chemical, and thermal stability; as well as desirable antifouling properties [86,87,88,89]. Until now, various advanced modification methods have been studied in order to improve the transport, mechanical, and antifouling properties of polymeric MF membranes [90,91,92]. In general, the modification techniques for MF membranes are classified in physical and chemical modification methods where the main difference between these two groups concerns the type of interactions on the surface or membrane bulk [90,91,93,94,95].

To date, several review articles have been published regarding MF membranes [96,97,98,99]. Urosevic et al. [100] prepared a review study regarding novel advancements in MF and UF membranes in the juice industry in 2017. The influence of crucial parameters—such as operating pressure, temperature, membrane type, and juice type—on the separation performance and juice quality was investigated. Several promising methods were proposed to mitigate membrane fouling in the juice industry, including employing cross-flow filtration mode, enzymatic pretreatment, mechanical, and electrical strategies [100]. In 2019, Anis et al. [101] provided a comprehensive review article concerning completed studies on MF membranes from 2009 to 2018. In that review, the authors provided statistical information on MF membrane publications across different fields, such as water purification, biotechnology, and the food industry. The review highlighted a significant growth in experimental studies, identifying modeling and optimization investigations as vital research gaps for overcoming the serious limitations (i.e., membrane fouling and flux decline) in MF membranes [101]. In 2021, Gul et al. [32] published a review article focusing on fouling phenomenon and cleaning techniques for reusing MF membranes. The review examined different types of membrane fouling (i.e., colloidal, organic, inorganic, and biofouling) together with their influence on the separation performance of MF membranes. A range of physical (i.e., forward and reverse flushing, air flushing, backwashing, relaxation, and sponge ball) and chemical cleaning (i.e., caustic, acids, alkalis, oxidants, enzymes, and surfactants) strategies were studied along with their benefits and drawbacks for restoring the MF membranes. Moreover, key cleaning parameters—such as cleaning time, cleaning operating temperature, and the concentration of cleaning agents—were studied for their impact on cleaning efficiency [32]. In 2022, Tanudjaja et al. [102] prepared a review article related to the protein-based fouling in MF and UF separation processes in the dairy, food, and wastewater treatment industries. The authors examined how the features of proteins can influence membrane fouling. Moreover, various promising methods for fouling reduction (protein-based) and the cleaning of fouled membranes were introduced and discussed in that review. It was found that two key factors were the main reasons for protein-based fouling: 1. interactions between proteins themselves and with the membrane surface; 2. the correlation between protein size and the average pore size of the membrane [102]. In 2025, Hu and Wang [103] published a review article concerning the mathematical models applied to MF membranes. The main goal of that review article was to introduce various mathematical models to optimize the performance of MF membranes for water and wastewater treatment. In that study, the benefits and drawbacks of each classical model were examined and compared to a recent artificial intelligence model. Moreover, the potential of a combination of classical models with an artificial intelligence model was explored [103].

**Figure 4:**
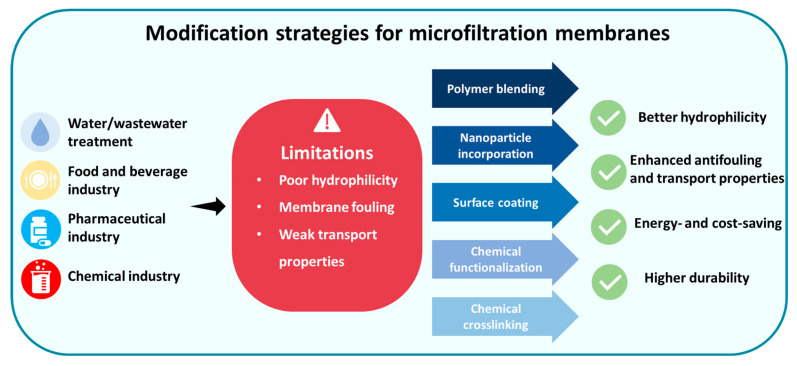
Modification strategies for polymeric MF membranes.

The main goals of most published review articles about MF membranes are related to membrane fouling and cleaning technologies for fouled membranes. Despite recent progress in the field, no review article has been published recently to address the novel physical (i.e., polymer blending, nanoparticle incorporation, amphilic copolymer integrating, plasma treatment, and thermal pressing/annealing) and chemical (i.e., chemical crosslinking, chemical surface functionalization, chemical grafting, and in situ nanoparticle immobilization) strategies for the modification and development of polymeric MF membranes (Figure 4). The primary objective of this review is to introduce innovative modification techniques (both physical and chemical methods) for optimizing the morphological, hydrophilic, and transport properties of polymeric MF membranes. The advantages and limitations of each method are well described accompanied by relevant examples from recent investigations. Eventually, valuable insights and approaches are introduced to achieve polymeric MF membranes with optimal functionality (i.e., fouling-resistant), service life, and mechanical stability.

## 2. Membrane Fouling Phenomenon

**Figure 5:**
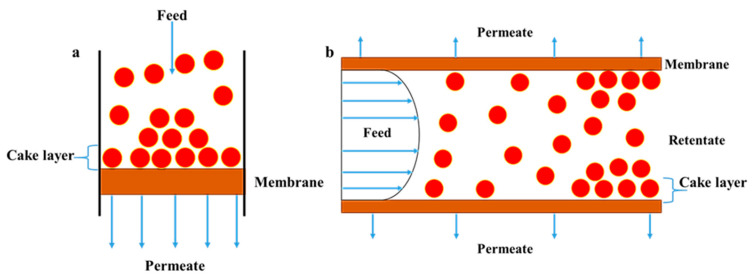
Formation of cake layer and membrane fouling in (**a**) dead-end and (**b**) cross-flow MF membranes.

The main limitation of MF membranes is membrane fouling, which occurs by the accumulation, deposition, or adsorption of unfavorable contaminants and particles on the surface of a membrane or within its structure, causing reduced flux and increased transmembrane pressure. This phenomenon generally happens through various mechanisms, such as pore blocking, cake layer formation, adsorption, and biofouling (Figure 5) [32,99]. These contaminants, including suspended solids, colloids, organic macromolecules, microorganisms, and inorganic precipitates, interact with a membrane’s surface during operation. Based on these interactions, two types of fouling can occur: reversible and irreversible fouling. In reversible fouling the interactions are governed by physical sieving, electrostatic attraction, hydrophobic interactions, while chemical bonding constitutes the principal cause of irreversible fouling [68,97,99].

**Figure 6:**
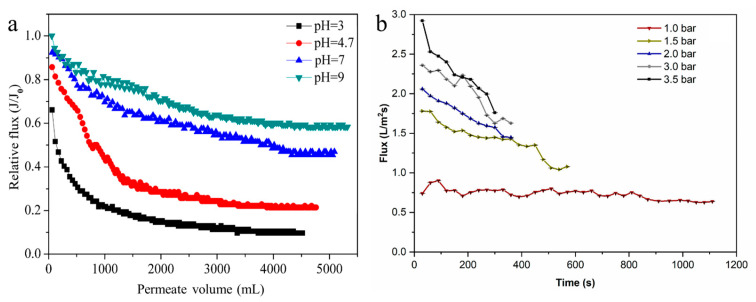
(**a**) Relative flux decline of the HA–BSA solution for the PES membrane at different pH values (adapted from Sun et al., 2018) [107]; (**b**) flux reduction in the synthetic cyanide solution over time for the PVDF membrane at different operating pressures (adapted from Quilaqueo et al., 2025) [57].

It should be noted that fouling phenomena significantly influence the performance of MF membranes (i.e., causes flux decline). Xiong et al. [104] evaluated the filtration performance of PVDF and polyethersulfone (PES) membranes containing different average pore sizes of 0.22 and 0.45 μm for the clarification of mulberry wine. It was found that the wine permeate flux after 60 min was reduced considerably between 55 and 66% owing to the cake layer formation and fouling phenomenon. In another research, Zhao and Chen [105] assessed the biofouling behavior of pure PVDF and composite PVDF-*g*-poly(hydroxyethyl methacrylate) (HEMA)-*co*-(*N*-isopropylacrylamide) (NIPAAm)) membranes employing bacterial suspensions as model foulants. It was found that flux reduction over time was between 8 and 70% for the PVDF-*g*-P(HEMA-*co*-NIPAAm) membrane, while a weaker performance was observed for the pure PVDF membrane (between 63 and 83%). Akamatsu et al. [106] investigated the filtration performance of polyethylene (PE) and surface-modified PE/poly(2-methoxyethyl acrylate) (PMEA) membranes in the sea water purification process. It was found that flux reduction over 24 h was ~19% for the surface-modified membrane, while a weaker performance was reported for the pure PE membrane (~34%). In another study, Sun et al. [107] evaluated the fouling behavior of a PES MF membrane during the filtration of a humic acid (HA)–bovine serum albumin (BSA) mixed solution. It was found that the HA–BSA relative flux was reduced considerably at different pH levels between 40 and 85% due to membrane fouling during the filtration process (Figure 6a). Similar observations were reported by Quilaqueo et al. [57], where the flux of the PVDF membrane was reduced over time at different operating pressures, while the highest flux decline values were reported at 3.5 and 3.0 bar (55 and 50%, respectively) because of fouling phenomenon (Figure 6b). The summarized information on fouling-induced flux decline from the mentioned studies is presented in Table 1.

The intensity of fouling phenomenon is significantly influenced by feed compositions and properties, operating conditions, and membrane characteristics. To minimize fouling phenomena in MF membrane processes, these parameters should be carefully optimized. In the case of feed compositions three important criteria should be taken into consideration: particle size, pH, and organic content. Having feed containing larger particles than the average pore size of the membrane causes the formation of a reversible cake layer, whereas fine particles can penetrate the membrane pores, causing irreversible pore blocking. Moreover, a lower organic content is desirable, since feed containing high concentrations of organic matter leads to increased adsorption onto the membrane surface. The pH should also be far from the isoelectric point (IEP) of the foulants to enhance repulsive interactions between contaminants and the membrane surface [98,99].

Operating conditions—flux, pressure, filtration type, and temperature—can strongly influence membrane fouling as well. Operating the MF process at low transmembrane pressure and below the critical flux is desirable, since when applying high pressure, contaminants and particles penetrate the pores and fill them (i.e., compacting the cake layer), causing irreversible fouling. Furthermore, membrane fouling can be controlled by employing cross-flow filtration, in which the feed flows parallel to the membrane surface. Such parallel flow creates shear forces that continuously sweep away contaminants from the membrane surface. The operating temperature also slightly affects membrane fouling, so a moderate temperature is recommended for MF operation because high temperatures may accelerate adsorption, bioactivity, and chemical interactions [32,102].

Another influential factor is optimizing membrane properties, such as pore size, surface roughness, and hydrophilicity. The pores should be considerably larger or smaller than the contaminants, since having pores and particles of similar sizes causes severe fouling. Additionally, a lower surface roughness is desirable because rougher surfaces contain gaps and valleys, which cause particle trapping and promote irreversible fouling. Another crucial parameter is related to the hydrophilic properties of MF membranes in which a higher hydrophilicity is favorable, since hydrophilic surfaces decrease hydrophobic interactions with organic particles and foulants (i.e., proteins) [32,97]. Table 2 provides information about the key factors influencing MF fouling and desirable conditions for weaker fouling.

## 3. Methods for Modification of Polymeric MF Membranes

**Figure 7:**
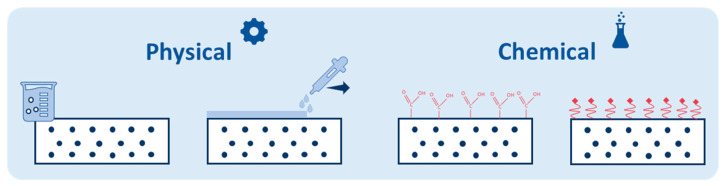
Schematic of physical and chemical modification methods for polymeric MF membranes.

Generally, the techniques for modifying polymeric MF membranes are classified into physical and chemical modification methods (Figure 7) [108,109,110,111]. The major difference between these two classifications concerns the type of interactions on the surface or membrane structure [111]. For the physical modification methods, there are only physical interactions on the surface and membrane structure (without chemical reaction and covalent bond) [108,109]. In contrast, chemical modifications involve strong interactions (i.e., covalent bonds) on the membrane structure (surface and inner structures) [111,112]. Therefore, chemical modification methods offer higher stability and effectiveness and are great choices for long-term applications (stable hydrophilic, transport, and antifouling properties) [111,112]. However, physical modification methods are easy, quick, inexpensive, highly scalable, less toxic, and usually reversible [113]. In the present review, both physical and chemical modification methods are explained together with their advantages and limitations accompanied by relevant recent examples.

### 3.1. Physical Modification Methods

This category of modification method offers several crucial benefits, such as low system complexity, cost-effectiveness, scalability, low risk, and reversibility. Nonetheless, the lack of stable transport and antifouling properties is the main limitation of this modification method. Polymer blending, nanomaterial incorporation, amphilic copolymer integration, surface coating, plasma treatment, thermal pressing/annealing, and the optimization of physical parameters are some of the most applicable physical modification methods for polymeric MF membranes [108,109,110,113,114,115,116,117,118].

**Figure 8:**
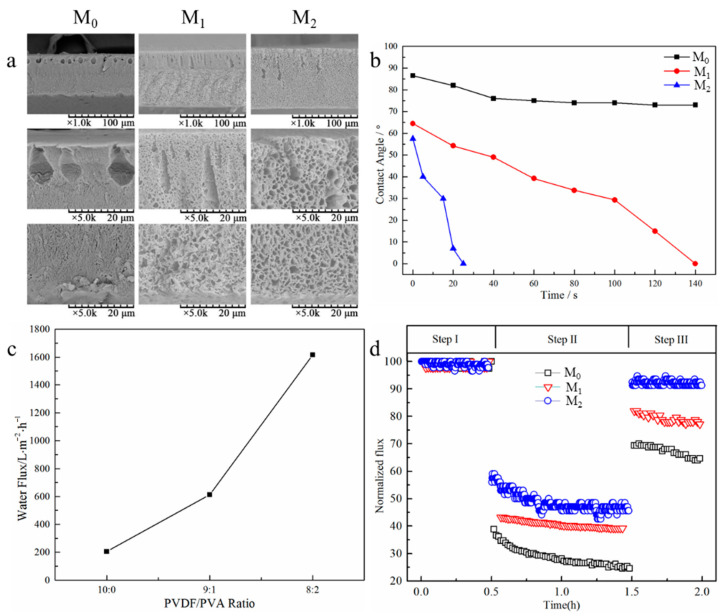
(**a**) Cross-section SEM images; (**b**) WCA over time; (**c**) pure water flux; (**d**) antifouling performance of the PVDF/PVA blend membranes (adapted from Hu et al., 2016) [119].

One of the most employed physical modification methods for polymeric MF membranes is polymer blending. In this method two or more polymers are directly blended in a common solvent. The prepared membranes by this method are often fabricated using the phase inversion method. This method is usually used to improve the morphological and/or hydrophilic characteristics of hydrophobic polymers (i.e., PVDF and polyethersulfone (PES)) by adding hydrophilic polymers, such as polyvinyl alcohol (PVA), chitosan, cellulose-based polymers (e.g., cellulose acetate, cellulose derivatives), polyetherimide (PEI), polyethylene glycol (PEG), and polyvinylpyrrolidone (PVP) [119]. It is worth mentioning that PVP and PEG can improve both the hydrophilicity of MF membranes, owing to the presence of hydrophilic groups in their structures, and their morphology, acting as pore-forming (porogens) agents during membrane preparation. Even though the polymer blending technique is scalable, simple, budget-friendly, and flexible, challenges related to dispersion and incompatibility are serious disadvantages of this method. Gunes-Durak [114] fabricated PS/polyetherimide (PEI) blend membranes containing various concentrations of polymers (i.e., 3.75, 7.50, 11.25, and 15 wt.%) via the non-solvent induced phase inversion (NIPS) method. The main objective of that research was to improve the structural and transport properties of PS membranes by introducing PEI. Water content (up to 16.73%), mean pore size (~390 nm), and porosity (up to 91.85%) values were enhanced for blend membranes compared with the pristine PS membrane (5.37%, 67.35 nm, and 30.61%, respectively). Moreover, blend membranes showed lower water contact angle (WCA) values (between ~67.75 and 78.24°) than pristine samples (~85.81°), confirming the improvement in the hydrophilicity of blend membranes. The pure water flux test showed higher values for blend PS/PEI membranes (up to ~342.17 L·m^−2^·h^−1^) in comparison with pristine PS membranes (~257.25 L·m^−2^·h^−1^). In addition, all blend membranes demonstrated a higher removal rate for various pollutants (i.e., sulfate, chloride, manganese, iron, and total organic carbon (TOC)) than PS membranes. Their research illustrates that pure water flux and the removal rate of PS/PEI membranes were enhanced by the blending method owing to the improvement of the hydrophilicity and morphology of membranes [114]. In another study, Hu et al. [119] fabricated porous PVDF/PVA blend membranes using the non-solvent thermally induced phase inversion (NTIPS) technique. Various blend membranes containing different PVDF/PVA blend ratios (PVDF/PVA ratio: 10:0 (M_0_), 9:1 (M_1_), and 8:2 (M_2_)) were prepared to evaluate the influence of PVA concentration on the hydrophilic, morphological, and transport properties of the fabricated membranes. Scanning electron microscopy (SEM) images demonstrated that by increasing PVA content, more porous membranes were obtained (Figure 8a). Moreover, membrane hydrophilicity was enhanced by increasing PVA content owing to the strong hydrophilic properties of this polymer (Figure 8b). It was found that the water flux improved significantly up to ~1600 L·m^−2^·h^−1^ for the M_2_ membrane compared with the pure PVDF membrane (~200 L·m^−2^·h^−1^) (Figure 8c). Additionally, the blend membranes (i.e., M_1_ and M_2_) exhibited a better antifouling performance than the pure PVDF membrane (Figure 8d). These observations reveal that the weak hydrophilic, transport, and antifouling properties of the PVDF membrane were enhanced considerably by adding PVA, owing to the presence of numerous hydroxyl groups in its structure.

**Figure 9:**
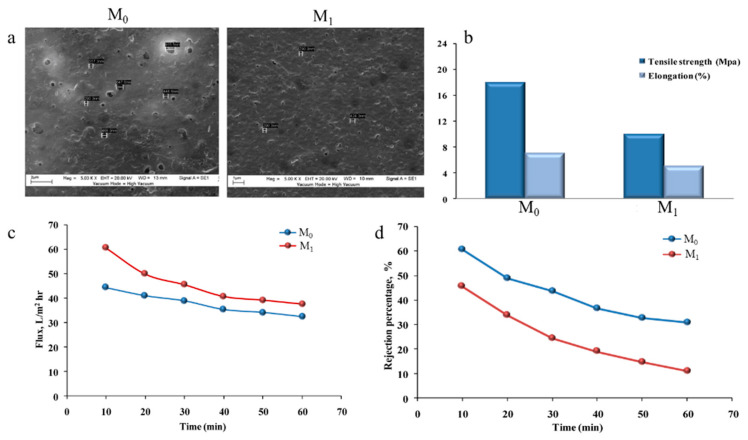
(**a**) SEM images (top surface); (**b**) mechanical properties; (**c**) phenol solution flux; (**d**) phenol rejection performance of the pure PS and PS/PEO blend membranes (adapted from Hussein et al., 2023) [120].

Hussein et al. [120] prepared MF blend membranes containing PS and polyethylene oxide (PEO) via the NIPS method to evaluate the mechanical stability, transport properties, and rejection performance of pure PS (M_0_) and PS/PEO blend (M_1_) membranes. The SEM images taken from the top surface of the membranes illustrated that by adding PEO, the apparent pore size of the membrane was reduced in comparison with the PS membrane (Figure 9a). In addition, the PS/PEO blend membrane exhibited a higher tensile strength and elongation at break values (~18 MPa and ~7%, respectively) than the pure membrane (~10 MPa and ~5%, respectively) (Figure 9b). It was observed that the pure PS membrane showed a higher water flux (~97 L·m^−2^·h^−1^) than the blend membrane (~73 L·m^−2^·h^−1^). A similar trend was observed for the phenol solution flux as well (Figure 9c). Furthermore, the M_1_ membrane (~60%) showed a better phenol rejection performance than the M_0_ membrane (~45%) (Figure 9d). Such observations confirm that by employing the polymer blending method, the morphological, mechanical, physical, and transport characteristics of an MF membrane can be changed significantly.

**Figure 10:**
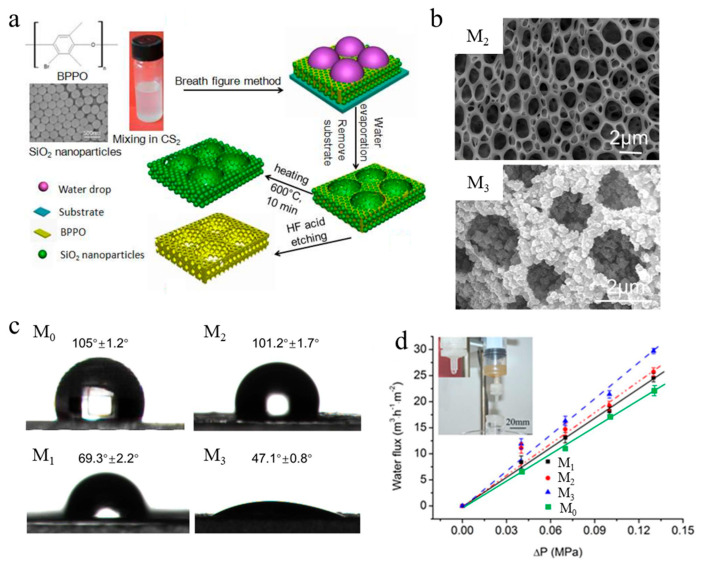
(**a**) Schematic of membrane fabrication; (**b**) SEM micrographs; (**c**) WCAs; (**d**) water flux of BPPO-based membranes (adapted from Yuan et al., 2018) [121].

Another well-known method is the incorporation of nanoparticles (NPs). In this method the composite membrane is prepared from a homogenous solution containing a polymer base and NPs. Such a method can significantly improve the hydrophilicity, mechanical stability, permeability, antimicrobial activity, and antifouling properties of membranes. However, the main drawbacks of this technique are related to the agglomeration and leakage of NPs as well as incompatibility issues. It should be mentioned that not all NPs employed in membrane preparation are inherently toxic; inorganic oxides such as SiO_2_ and certain biopolymer-based NPs are generally considered low-toxicity nanomaterials. Conversely, the main concern has been associated with some metal and metal oxide NPs, such as silver (AgNPs), zinc oxide (ZnO), copper and copper oxide (Cu and CuO), and titanium dioxide (TiO_2_) NPs, owing to their potential environmental and health impacts, especially in the event of NP release. As a result, appropriate safety measures are required when handling these NPs, as exposure through inhalation or dermal contact may pose health risks. A diverse array of NPs has been employed in MF separation processes, including TiO_2_, SiO_2_, ZnO, graphene oxide (GO), carbon nanoparticles (CNPs), and AgNPs. El Jery et al. [113] developed a poly(acrylonitrile-styrene) (SAN) MF membrane using AgNP incorporation. The primary goal of such a choice was to benefit from the antibacterial and antifouling features of AgNPs. Several membranes possessing different contents of AgNPs (0, 1, 1.5, 2, 2.5, 3, 4, 10 g) were prepared. It was found that the porosity and pore size of the composite membranes decreased initially by increasing the content of NPs and then increased compared with the unmodified SAN membrane (~85% and 3.5 μm, respectively). These observations could be due to the accumulation of AgNPs causing changes in morphological properties. Moreover, SAN/AgNPs membranes showed better antibacterial properties (94 to 98%) than an unmodified membrane. The composite membrane containing 4 g of AgNPs demonstrated a higher water flux (511 L·m^−2^·h^−1^) than the unmodified SAN membrane (~360 L·m^−2^·h^−1^). Additionally, composite membranes exhibited higher flux recovery ratios (FFRs) up to 94.9% in comparison with the unmodified membrane (~73%). Such observations confirm the enhancement of the transport, antibacterial, and antifouling properties of an SAN/AgNPs composite membrane as the incorporation of AgNPs enhanced the morphological (i.e., porosity and pore size) and antibacterial properties of the SAN membrane [113]. In another study, Yuan et al. [121] fabricated porous MF membranes containing brominated polyphenylene oxide (BPPO) and SiO_2_ NPs using the breath figure method. The work involved fabricating four membranes with different structures: a porous BPPO membrane (M_0_), a BPPO/SiO_2_ composite membrane (M_1_), a hierarchical porous polymer (HPP) membrane (M_2_) prepared by removing the SiO_2_ NPs from M_1_, and a BPPO/SiO_2_–C membrane (M_3_) fabricated by carbonizing the M_1_ membrane (Figure 10a). SEM micrographs demonstrated a heterogeneous porous structure for BPPO-based membranes and showed the uniform distribution of SiO_2_ NPs in the membrane structure (Figure 10b). Moreover, water contact angle (WCA) measurements showed that the addition of SiO_2_ NPs enhanced membrane hydrophilicity owing to the excellent hydrophilic properties of SiO_2_ (i.e., the presence of SiOH chains) (Figure 10c). Furthermore, the BPPO/SiO_2_–C composite membrane (M_3_) exhibited the highest water flux of ~30 × 10^3^ L·h^−1^·m^−2^, confirming that thermal treatment of BPPO/SiO_2_ membrane led to improved membrane morphology (Figure 10d). These results show that the addition of SiO_2_ NPs and the thermal treatment of the resulting membranes contributed to enhanced hydrophilic and transport properties of the MF membranes.

Severcan et al. [122] prepared PS/polyethylenimine (PEI)-based composite membranes modified by TiO_2_ and Al_2_O_3_ NPs to improve pomegranate juice clarification efficiency. It was found that the PS/PEI/TiO_2_ and PS/PEI/Al_2_O_3_ composite membranes demonstrated higher porosity values (~80 to 84% and ~77 to 85%, respectively) than the PS membranes (~69%). Additionally, the composite membranes showed lower WCA values (between 69 and 86°) in comparison with the pure PS membrane (94°) due to the strong hydrophilic character of PEI and NPs. The water flux measurements showed a weak performance for the pure PS membrane (135 L·m^−2^·h^−1^), while the flux increased significantly for PS/PEI/TiO_2_ and PS/PEI/Al_2_O_3_ composite membranes (~2800 and 1500 L·m^−2^·h^−1^, respectively). Such observations can be explained by the higher porosity and hydrophilicity of the composite membranes compared with the pure membrane. Furthermore, the composite membrane exhibited an excellent performance in turbidity reduction (99.9%). These observations confirm that the incorporation of NPs can considerably enhance the morphological, hydrophilic, and transport characteristics of MF membranes.

Another strategy to modify the transport and antifouling properties of porous membranes (i.e., UF and MF) is amphilic copolymer integration. These types of copolymers are able to improve the hydrophilicity of hydrophobic membranes, such as PVDF and PES, owing to the presence of hydrophilic segments in their structure. The main challenge of this method is related to the compatibility between the polymer base and the selected amphilic copolymer, for which the presence of hydrophobic segments can reduce the risk of incompatibility. Wang and co-workers [115] prepared blend membranes possessing PES and poly(poly(ethyleneglycol) methylethermethacrylate (MAPEG)-*co*-2-(methacryloyloxy)ethyl 5-(1,2-dithiolan-3-yl)pentanoate (MALA)-*co*-(4-formylphenyl)-2-methylprop-2-enoate (MAHA)) using the phase inversion technique. The main reason for choosing such a copolymer was to overcome the main downside of PES which is an inherently hydrophobic polymer, causing membrane fouling and flux reduction. PES/P(MAPEG-*co*-MALA-*co*-MAHA) membranes showed higher porosity (between 37 and 43%) and pore size values (between 142 and 163 nm) in comparison with abare PES membrane (36% and 139 nm, respectively). Moreover, blend membranes showed lower WCA values (to approximately 54°) than a bare membrane (~74°), reflecting the improvement of membrane hydrophilicity. It was found that PES/P(MAPEG-*co*-MALA-*co*-MAHA) membranes demonstrated higher pure water flux (~89 L·m^−2^·h^−1^) and FRR (within the interval 89.9–93.3%) values compared with the PES membrane (~39 L·m^−2^·h^−1^ and 68.3%, respectively). Such observations confirm the enhancement of the morphological, hydrophilic, transport, and antifouling properties of modified membranes in comparison with a bare PES membrane owing to the introduction of an amphilic copolymer (containing excellent hydrophilic features) [115].

**Figure 11:**
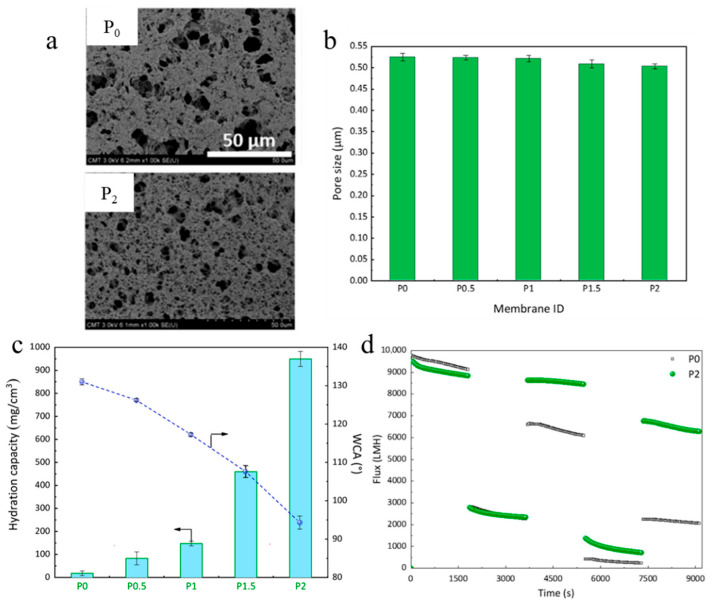
(**a**) SEM micrographs; (**b**) membrane pore size; (**c**) WCA and hydration capacity; (**d**) water/bacteria filtration performance of PVDF and PVDF/P(S-*r*-EGMA) membranes (adapted from Aini et al., 2022) [123].

In another study, Aini et al. [123] fabricated porous MF membranes containing PVDF and poly(styrene-*r*-(ethylene glycol) methyl ether methacrylate) (P(S-*r*-EGMA)) using the vapor-induced phase inversion (VIPS) technique. Different membranes containing various concentrations of P(S-*r*-EGMA) (0 (P_0_), 0.5 (P_0.5_), 1 (P_1_), 1.5 (P_1.5_), and 2 (P_2_) wt.%) were prepared to evaluate the influence of P(S-*r*-EGMA) concentration on the hydrophilic, morphological, and transport properties of the fabricated membranes. The addition of copolymers led to a slight decrease in pore size and porosity values compared with the pure PVDF membrane, which can be attributed to the larger volume fraction occupied by P(S-*r*-EGMA) (Figure 11a,b). However, PVDF/P(S-*r*-EGMA) membranes showed higher hydration capacity and lower WCA values than the pure PVDF membrane, revealing an improved hydrophilicity of the modified membranes owing to the excellent hydrophilic nature of PEGMA (Figure 11c). Moreover, it can be seen that the P_2_ membrane, due to its high hydrophilicity, showed a better water/bacteria filtration performance than the P_0_ membrane (Figure 11d). Overall, these results clearly highlight the positive effect of copolymer addition on the hydrophilic and transport properties of PVDF MF membranes.

**Figure 12:**
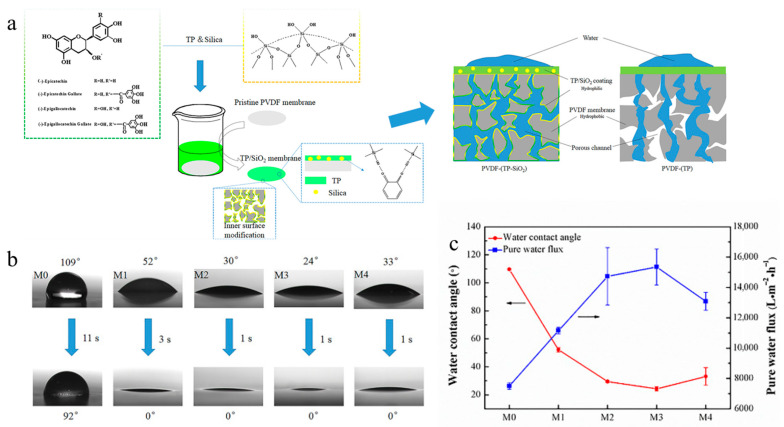
(**a**) Coating schematic diagram; (**b**) WCA; (**c**) pure water flux of PVDF/TP-silica composite membranes (adapted from Xu et al., 2021) [116].

Surface modification is another effective physical modification method to improve the morphological, hydrophilic, and transport characteristics of porous membranes (i.e., MF, UF, and NF). Physical surface modification can be carried out using different approaches, including dip coating (sample immersion), spray coating, filtration-based coating, physical deposition, and layer-by-layer assembly techniques. Despite the fact that this modification method is not too costly and complex, such membranes often do not make excellent candidates for long-term applications owing to their lack of chemical bonds (i.e., covalent). Xu et al. [116] fabricated PVDF surface-modified membranes by coating a tea polyphenols (TPs)–silica composite onto the surface and inner structure of membranes (Figure 12a). The chief aim of that research was to improve the hydrophilicity of a PVDF membrane with a hydrophobic nature. Scanning electron microscopy (SEM) images demonstrated a homogenous top layer structure for the pure PVDF membrane. However, the top layer structure changed to heterogenous for surface-modified membranes. Surface-modified membranes showed lower values of WCA (between 24 and 52°) compared with the pure PVDF membrane (~109°), verifying the improvement of the surface hydrophilicity of membranes (Figure 12b). Moreover, the water flux of surface-modified membranes (15,353 L·m^−2^·h^−1^) was higher (approximately two times) than a pure PVDF membrane (Figure 12c). The emulsion rejection rate of modified membranes was enhanced up to 97%; such a performance was better than the pure PVDF membrane (~94%). These results clearly demonstrate the positive impact of the tea polyphenols/silica composite coating on the morphological, hydrophilic, and transport properties of surface-modified membranes [116].

**Figure 13:**
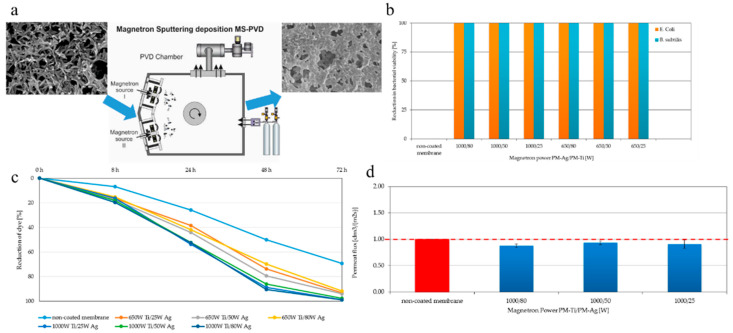
(**a**) Coating schematic diagram; (**b**) bacterial rejection; (**c**) dye rejection performance; (**d**) water permeate flux of polyamide-based membranes (adapted from Kacprzyńska-Gołacka et al., 2021) [91].

In another study, Kacprzyńska-Gołacka et al. [91] fabricated surface-modified membranes via TiO_2_-AgO coating on polyamide (PA) MF membranes. The surface coating was performed using the magnetron sputtering technique at different magnetron powers (25–1000 W). SEM micrographs revealed a uniform coating on the surface of PA membranes, even though some agglomerations were observed as well (Figure 13a). It was found that all surface-modified membranes showed a high bacterial rejection ability of 100% (for both *Escherichia coli* and *Bacillus subtilis*), confirming the great antibacterial performance of surface-modified membranes (Figure 13b). Surface-modified membranes showed higher methylene blue (MB) rejection (over 90%) than a pure PA membrane (~68%) after 72 h (Figure 13c). However, the pure PA membrane exhibited a slightly higher water permeate flux than the modified membranes, owing to pore coverage by the TiO_2_-AgO, which led to a denser surface structure (Figure 13d). These findings indicate that membranes with an enhanced bacterial and dye rejection performance can be obtained by applying a TiO_2_-AgO coating onto the surface of a PA membrane, although this modification slightly decreases water flux.

Plasma treatment is another useful technique for improving membrane morphology (surface roughness) and hydrophilicity (wettability). In this physical modification method, well-known polar functional groups, such as hydroxyl, carboxyl, and amino groups, were introduced onto the membrane surface. Therefore, the membrane surface becomes functionalized, which makes it more hydrophilic, contributing to better antifouling properties. This physical modification method is considered an environmentally friendly process because no new chemicals are added during the modification process. One of the negative features of this method is the gradient of properties since only the membrane surface is treated and the membrane bulk (i.e., inner structure) cannot be modified. Moreover, exposure time should be optimized, otherwise the membrane surface will be damaged. Afkham et al. [117] fabricated and modified PES-based MF membranes using the plasma technique. The core purpose of such modification was to overcome the hydrophobic properties of a PES membrane causing flux decline and fouling phenomenon by applying corona plasma treatment. The MF membranes were prepared using VIPS in combination with the NIPS method. In that study, the influence of input power (300, 500, and 700 W) and exposure time (0, 7, 10, and 13 min) of plasma treatment on the morphological, hydrophilic and transport properties of PES membranes was evaluated. It was found that the surface roughness of modified membranes reduced (in the range of 189–440 nm) compared with the unmodified PES membrane (~507 nm). No significant change in porosity was observed, while the average pore size of modified membranes declined to 210 nm in comparison with the unmodified membrane (~302 nm). In addition, the plasma treatment led to a water contact angle reduction from ~60° for unmodified PES membrane to ~10–20° for plasma treated membranes, confirming better membrane hydrophilicity and wettability. The highest water and milk flux values (16,000 and 13.2 L⸱m^−2^⸱h^−1^, respectively) were reported for the modified membrane with 300 W applied power and 10 min exposure time. All treated membranes revealed a higher microbe rejection ability (between 74.1 and 92.4%) than an unmodified membrane (54.6%). Moreover, all modified membranes demonstrated higher FFR values (from 39.5 to 86.5%) than an unmodified PES membrane (36.7%). The findings indicate that, owing to the introduction of polar functional groups on the surface of membranes using the plasma treatment technique, the morphological, hydrophilic, transport, and antifouling properties of modified membranes improved in comparison with an unmodified PES membrane [117]. Moreover, fine particles can be coated onto the surface of MF membranes using the plasma treatment method.

**Figure 14:**
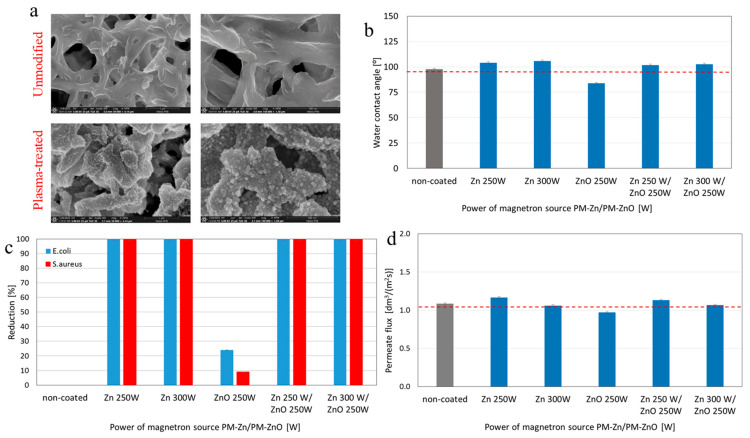
(**a**) SEM micrographs; (**b**) WCA; (**c**) bacterial rejection; (**d**) water permeate flux of PA-based membranes (adapted from Kacprzyńska-Gołacka et al., 2023) [124].

Kacprzyńska-Gołacka et al. [124] prepared surface-modified membranes by applying Zn, ZnO, and Zn-ZnO coatings on PA MF membranes. The surface coating was performed using the plasma treatment method at different power levels (250–300 W). SEM micrographs showed a uniform coating on the surface of PA membranes, although some agglomerations were seen (Figure 14a). Among all plasma-treated membranes, only the PA/ZnO membrane showed a lower WCA value (~84°) than the unmodified PA membrane (~98°), confirming the excellent hydrophilic properties of ZnO (Figure 14b). Unlike the unmodified PA membrane, the plasma-treated membranes showed a high bacterial rejection ability (up to100%) (for both *Escherichia coli* and *Staphylococcus aureus*), confirming the excellent antibacterial performance (Figure 14c). Furthermore, plasma-treated membranes exhibited a higher water permeate flux than the unmodified PA membrane (Figure 14d). These findings confirm that the plasma treatment method can considerably enhance the morphological, transport, and antibacterial properties of MF membranes.

**Figure 15:**
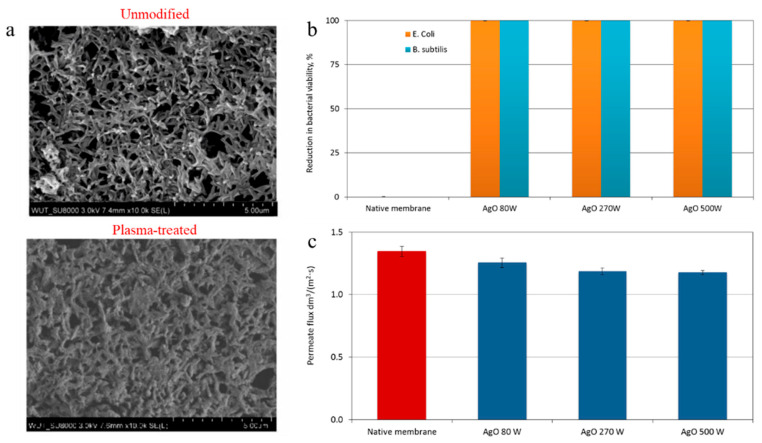
(**a**) SEM micrographs; (**b**) bacterial rejection; (**c**) water permeate flux of PA-based membranes (adapted from Kacprzyńska-Gołacka et al., 2020) [125].

In another study, Kacprzyńska-Gołacka et al. [125] fabricated surface-modified membranes by applying AgO coatings to PA membranes. The surface coating was carried out employing the plasma treatment strategy at various power levels (80–500 W). SEM images illustrated a uniform coating on the surface of PA membranes, despite the fact that some AgO accumulations were observed (Figure 15a). Unlike the unmodified membrane, the plasma-treated membranes showed a promising bacterial rejection ability (~99.9%) (for both *Escherichia coli* and *Bacillus subtilis*), revealing a great antibacterial performance (Figure 15b). Nonetheless, the unmodified membrane showed a slightly higher water permeate flux than the plasma-treated membranes, because of pore coverage by the AgO, which resulted in a denser surface structure (Figure 15c). These observations indicate that membranes with an improved bacterial performance can be obtained by applying the plasma treatment method even though this modification slightly decreased water permeate flux.

**Figure 16:**
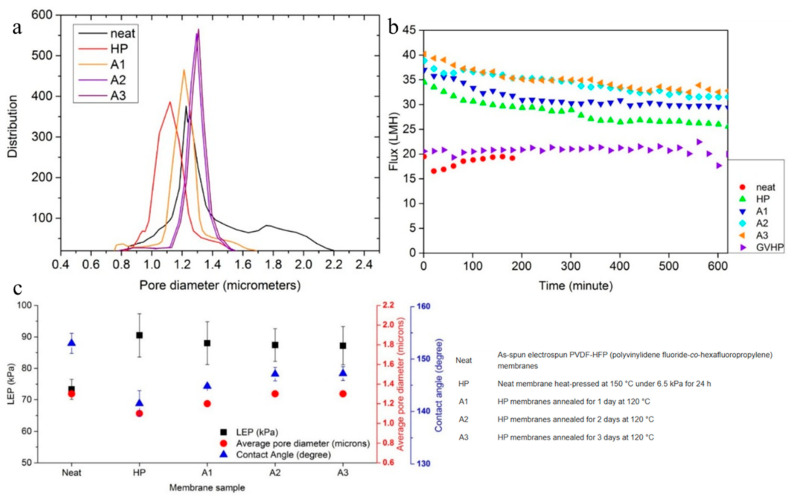
(**a**) Pore diameter distribution; (**b**) NaCl solution flux; (**c**) LEP of PVDF-*co*HFP-based membranes (adapted from Yao et al., 2017) [118].

The thermal pressing/annealing (heat treatment) method is another useful physical modification method for polymeric porous membranes. This method is a post-treatment technique where the polymeric membrane is initially fabricated (by phase inversion or electrospinning). Subsequently, the prepared membrane is heated under vacuum or atmospheric environment at a certain temperature for specified duration. By applying heat, the membrane’s morphology and structure change considerably. Such morphological changes (i.e., pore size and porosity) can notably affect the membrane’s permeability and selectivity. This modification method is considered an eco-friendly strategy since no chemical compounds are used. However, overheating can compress and close the pores and may even cause the thermal decomposition of the membranes. Yao and colleagues [118] prepared MF polyvinylidene fluoride-*co*-hexafluoropropylene (PVDF-*co*-HFP) membranes using the electrospinning technique for employment in the membrane distillation (MD) process. The obtained membranes were modified by the thermal pressing/annealing strategy for improving the low liquid entry pressure (LEP). Such thermal modification can improve the morphological and transport properties of membranes. The post-modification method was divided into two steps: 1. heat pressing (at 150 °C for 24 h); 2. thermal annealing (at 120 °C for between 1 and 3 days). The electrospun PVDF-*co*-HFP membrane showed a broad pore distribution within the interval 0.8–2.2 μm. The thermal pressing/annealing modification steps led to narrower pore size distribution which meant that membranes with a mean pore size between 1.1 and 1.3 μm were obtained (Figure 16a). The post-treated membranes revealed an excellent NaCl rejection of 99.99%. Furthermore, the heat-pressed (up to 28.7 L·m^−2^·h^−1^) and annealed (up to 35 L·m^−2^·h^−1^) membranes showed higher permeation fluxes than a pure electrospun PVDF-*co*-HFP membrane owing to its immediate wetting (Figure 16b). It was found that the modified membrane showed higher LEP values (~91 kPa) than the pure PVDF-HFP membrane (~73 kPa) (Figure 16c), verifying the better wetting resistance and MD stability of thermally treated membranes [118].

Arribas et al. [66] fabricated thermal-treated PS membranes employing a heat post-treatment strategy. In that study, PS nanofibrous membranes were initially prepared utilizing the electrospinning technique. Subsequently, the electrospun membranes were heated at different operating temperatures (210, 220, and 230 °C) and for different times (45, 60, 75, 90, 120, 150, 180 min). The SEM images showed that the rise in operating temperature or time resulted in a reduction in the mean size of the inter-fiber space and the void volume fraction, while increasing the mean nanofiber diameter. Moreover, the WCA demonstrated downward tendencies by increasing operating temperature and time. The humic acid (HA) flux reduced significantly with increasing heat post-treatment temperature and time, whereas the irreversible fouling and HA separation factors were enhanced. Indeed, increasing both the heat post-treatment temperature and time changed the membrane morphology, leading to denser membranes. The thermal-treated membranes showed a higher HA flux (133 kg·m^−2^·h^−1^) than a commercial PES membrane (82 kg·m^−2^·h^−1^), confirming their strong potential of these membranes in water treatment applications.

**Figure 17:**
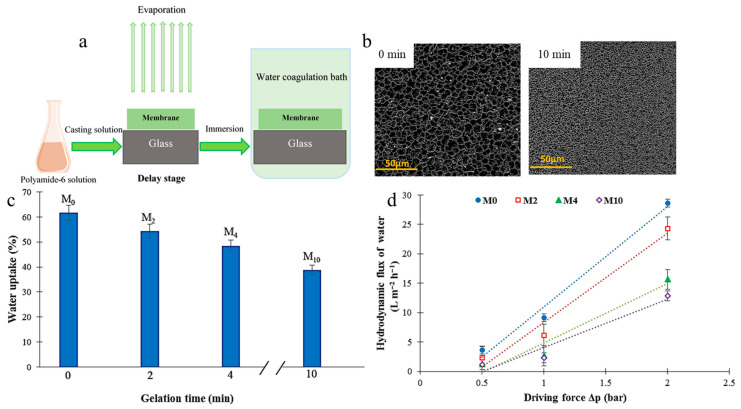
(**a**) Schematic of membrane fabrication; (**b**) SEM images; (**c**) water uptake; (**d**) water flux of PA6-based membranes (adapted from Ebrahimi et al., 2022) [31].

Another interesting strategy for the physical modification of porous membranes (i.e., MF, UF, and NF) is the optimization of physical parameters, such as the composition of coagulation media, the temperature of coagulation media, membrane casting rate, and gelation time. These physical parameters can directly influence and change the morphological, structural, and mechanical properties (i.e., porosity, pore size, swelling ratio, surface roughness, as well as water uptake) of MF membranes. Such changes in properties can significantly affect the transport and antifouling properties of porous membranes. These types of modification methods are easier, more cost-effective, and more environmentally friendly than the mentioned physical modification methods. Nonetheless, the positive influence of such modifications is not as significant as the physical modification methods mentioned (i.e., polymer blending, NPs incorporation, amphilic copolymer integrating, and surface modification). Ebrahimi et al. [31] fabricated PA6 MF membranes with different gelation times (0 (M_0_), 2 (M_2_), 4 (M_4_), and 10 (M_10_) min) using the NIPS technique (Figure 17a). The main objective of that research was to modify the morphological, structural, and mechanical characteristics of PA6-based membranes by changing gelation time. SEM images illustrated that the rise in gelation time led a decrease in the apparent pore size of membranes. Such behavior was explained by the fact that during the increase in the gelation time from 0 to 10 min, a larger amount of the volatile solvent (formic and acetic acid) evaporated from the cast polymeric film. Thus, changes in the skin layer of the M_10_ membrane were more visible than the M_0_ membrane (Figure 17b). Moreover, the M_0_ membrane showed the highest values of porosity (72%), mean pore size (0.166 μm), and water uptake (62%) while M_10_ revealed the lowest values (39%, 0.234 μm, and 39%, respectively) (Figure 17c). The increase in gelation time resulted in the enhancement of tensile strength up to 4.1 MPa. The M_0_ membrane demonstrated the highest pure water flux of 28.6 L·m^−2^·h^−1^ compared with the M_10_ sample (12.9 L·m^−2^·h^−1^) whereas the M_10_ membrane showed the highest PEG rejection value of ~50% compared with the M_0_ membrane (Figure 17d). Such research illustrates that only by optimizing one physical parameter (i.e., gelation time), the morphological, structural, mechanical, and transport properties of the membrane are considerably changed [31].

Vandezande et al. [126] prepared porous polyimide (PI) membranes via the NIPS method. In that study, the effects of fabrication parameters—such as polymer concentration and evaporation time—on the morphological and transport properties, as well as the rejection performance of PI-based membranes, were investigated. It was found that the increase in PI concentration from 15 to 25 wt.% led to a decrease in 2-propanol (2-PrOH)-based solution permeance, while increasing Rose Bengal (RB) rejection (up to 99.7%). In fact, by increasing the polymer concentration, less porous membranes (i.e., denser structures) were obtained, leading to flux reduction but enhanced rejection. Furthermore, similar trends were observed when increasing the evaporation time from 0 to 120 s (i.e., lower flux and higher RB rejection). The increase in evaporation time resulted in the greater evaporation of the volatile solvent, leading to densification (i.e., altering skin–layer morphology from a porous to a dense structure) and decreased flux. These findings indicate that by choosing optimal physical fabrication parameters, the morphological, transport, and rejection performance of MF membranes can be considerably enhanced. Table 3 presents an overview of the merits and demerits of different physical modification methods for MF membranes. Table 4 provides a summary concerning the various physical modification methods for MF membranes.

### 3.2. Chemical Modification Methods

In this class of modification methods for polymeric MF membranes, the functionality of membranes improves. Unlike physical modification methods, the surface and structure of membranes are permanently changed owing to strong chemical bonds (i.e., covalent bonds). Such modified membranes can offer more stable transport and antifouling properties compared with physically modified membranes. However, these modifications are usually complex, time-intensive, and expensive. There are some crucial chemical modification methods for MF membranes, namely crosslinking, chemical surface functionalization, chemical grafting, chemical surface modification, and in situ nanoparticle immobilization [111,112,127,128,129,130,131,132].

**Figure 18:**
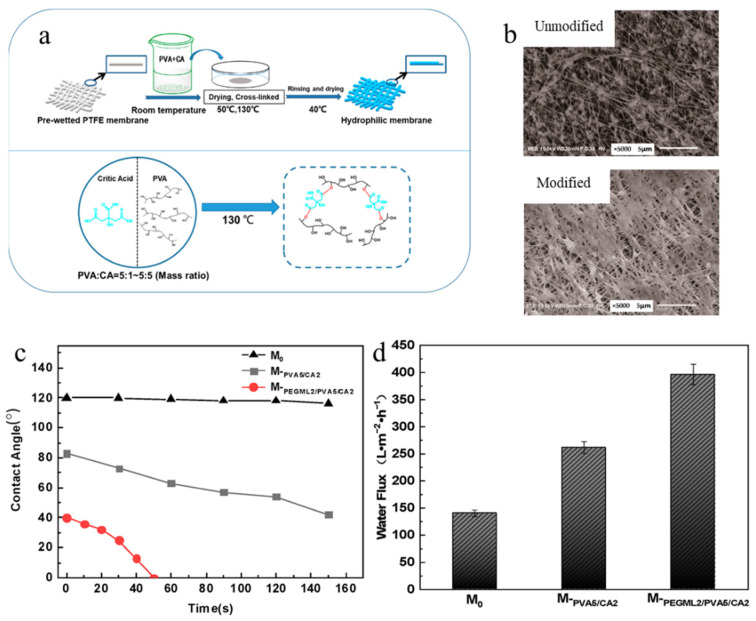
(**a**) Schematic of membrane modification; (**b**) SEM micrograph; (**c**) WCA; (**d**) water flux of PTFE-based MF membranes (adapted from Xu et al., 2022) [133].

One of the most commonly employed chemical modification methods for polymeric MF membranes is crosslinking reactions. In this method, polymer chains are chemically bonded together or with additives. For this reason, in this modification method, different crosslinkers are widely used to form chemical bonds, including glutaraldehyde, diisocyanates, epichlorohydrin, and citric acid (which is more environmentally friendly). These crosslinkers can properly react with hydroxyl and amine groups. As is expected, crosslinked membranes can provide stable transport and antifouling properties. However, such a method may contain several complex chemical reactions together with challenging control and operating conditions. Additionally, this method has negative impacts on the ecosystem owing to the chemical reactions and the addition of crosslinkers. Ong et al. [127] modified a polycarbonate track-etched (PCTE) MF membrane using the crosslinking strategy in order to enhance its resistance to organic solvents while maintaining satisfactory morphological, hydrophilic, mechanical, and transport properties. The first step of membrane modification was to immerse MF membranes in the solution containing branched polyethylenimine (BPEI) and water leading to the formation of bonds between amine and carbonate groups. Then, the crosslinking reaction was performed by the immersion of the PCTE/BPEI membrane in a solution containing 1,4-butanediol diglycidyl ether (BDG, crosslinker) to prepare a PCTE/BDG/BPEI membrane. It was found that the mean pore size and porosity values of the crosslinked membrane (~1.75 μm and 10.83%) were slightly reduced compared with the pristine membrane (~1.77 and 14.33%, respectively). However, the WCA value was slightly increased in the case of the crosslinked membrane (due to a decline in hydrophilicity) owing to the crosslinking stage (urethane bonds). The chemically modified membrane revealed very low swelling degrees (ranging from 1 to 6.2%) in various solvents, confirming the excellent solvent resistance of the PCTE/BDG/BPEI membrane against organic solvents. Moreover, the crosslinked membrane exhibited higher tensile strength (35.86 MPa) and Young’s modulus (3.50 MPa) values in comparison to the pristine membrane (27.96 and 2.94 MPa, respectively). However, the pristine PCTE membrane demonstrated a higher water permeance (~1050 L⸱m^−2^⸱h^−1^·bar^−1^) than the crosslinked sample (~1000 L⸱m^−2^⸱h^−1^·bar^−1^). The findings indicate that although the crosslinking reaction caused a slight decrease in the hydrophilic and transport properties of PCTE/BDG/BPEI membrane, the chemically crosslinked membrane exhibited great resistance to different organic solvents [127]. In another study, Xu et al. [133] modified the morphological, transport, and antifouling features of porous polytetrafluoroethylene (PTFE) MF membranes by combining surfactant bilayer assembly with a crosslinked hydrophilic coating. Initially, a polyethylene glycol laurate (PEGML) layer was formed on the PTFE membrane. Subsequently, a second layer consisting of crosslinked PVA and citric acid (CA) was deposited (Figure 18a). The SEM images showed a uniform formation of a hydrophilic layer on the PTFE/PEGML/PVA/CA membrane. However, the modified membranes showed a lower apparent pore size than the unmodified PTFE membrane (Figure 18b). It can be seen that the WCA of the modified membranes reduced significantly (between 42 and 83°) in comparison with the unmodified PTFE membrane (~120°), confirming the enhanced hydrophilicity of the crosslinked membranes, owing to the strong hydrophilic nature of PEGML and PVA (Figure 18c). Additionally, the PTFE/PEGML/PVA/CA membrane showed a higher water flux of 397 L·m^−2^·h^−1^ compared with the unmodified membrane (130 L·m^−2^·h^−1^) (Figure 18d). Furthermore, the modified membrane exhibited a higher flux recovery ratio (64%) than the unmodified membrane (44%), confirming the enhanced antifouling performance of the modified membrane.

This modification method can be combined with other methods, such as surface coating. Verma and Subbiah [128] chemically modified a polypropylene (PP) MF membrane using the coating/crosslinking method. The main goal of that research was to improve the antifouling properties of a PP membrane through the chemical deposition of sericin (possessing hydroxyl, carboxyl, and amino groups) on the membrane surface. The chemical modification of PP membranes was carried out in two main steps. In the first step, the sericin was coated onto the surface of the membrane using a flow-through strategy. In the next step (crosslinking step), the coated membrane was immersed into a solution containing glutaraldehyde (GA, crosslinker) and sulfuric acid; this essential step efficiently prevented sericin leakage from the membrane’s surface. The modified membrane showed a higher water content of 64.87% than the unmodified membrane (50.29%) while a lower porosity value (35.16%) was reported for the modified membrane compared with the unmodified one (53.63%). Moreover, the modified membrane showed a lower WCA value (~38°) in comparison with the unmodified PP membrane (~81.5°), confirming the better hydrophilic properties of the chemically modified membrane owing to the excellent hydrophilic character of sericin. However, such modification caused a pore size reduction in the modified membranes, resulting in a pure water flux reduction in the modified membranes (between 6.66 and 7.57 L⸱m^−2^⸱h^−1^) compared with the unmodified PP membrane (7.97 L⸱m^−2^⸱h^−1^). The membrane fouling was mitigated by 15% for the chemically modified membranes compared with the unmodified one during a gray water treatment test. That study illustrates that the hydrophilic and antifouling properties of a PP membrane were enhanced by the chemical coating/crosslinking method [128].

**Figure 19:**
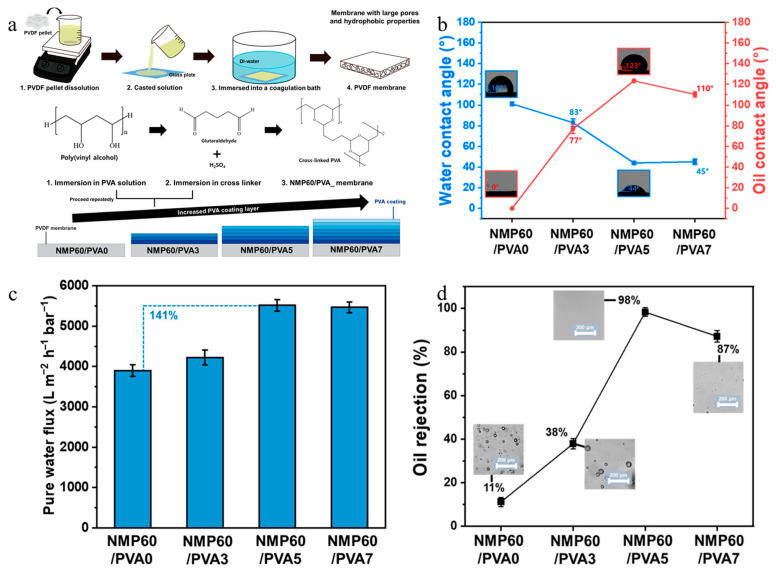
(**a**) Schematic of membrane modification; (**b**) water and oil contact angles; (**c**) pure water flux; (**d**) oil rejection performance of PVDF-based MF membranes (adapted from Choi and Nam, 2025) [134].

In another study, Choi and Nam [134] prepared porous PVDF membranes by depositing PVA layers for oily wastewater treatment. For this reason, they prepared initially porous MF membranes using the NIPS method. Subsequently, to improve the hydrophilic properties of hydrophobic PVDF, several PVA layers were deposited on the membrane surface. The crosslinked membranes were fabricated by dip-coating the PVDF membranes in a polymeric solution containing PVA, glutaraldehyde (the cross-linker), and sulfuric acid. This process was repeated three (PVDF/PVA_3_), five (PVDF/PVA_5_), and seven (PVDF/PVA_7_) times to assess the influence of the PVA layer on the membranes’ performance (Figure 19a). It was observed that the WCAs of crosslinked membranes reduced significantly (to between 45 and 83°) compared with the pure PVDF membrane (101°) (Figure 19b), confirming the enhanced hydrophilicity of the modified membranes. Moreover, the pure water flux of the modified PVDF/PVA membranes increased by around 141% in comparison with the pure PVDF membrane due to the improved hydrophilic properties of the crosslinked membranes (Figure 19c). In addition, the modified membranes exhibited higher oil rejection values (between 38 and 98%) compared with the pure PVDF membrane (11%) (Figure 19d). Overall, these observations confirm the enhancement of the hydrophilic, transport, and rejection performance of the PVDF membrane achieved by depositing crosslinked layers onto it.

Another well-known chemical modification approach for polymeric MF membranes is the chemical surface functionalization method. Generally, the main target of such a modification method is the membrane surface, for which the hydrophilic, transport, antifouling and antibacterial features of MF membranes are improved by performing chemical reactions on the surface of the membrane. Even though this modification method is mainly durable, stable, and customizable, process complexity, scalability issues, flux decline, high cost, and environmental concerns are the main disadvantages of this method. Yu and colleagues [129] modified a polyamide (PA) MF membrane via the chemical surface functionalization method to disinfect water. The primary objective of that research was to enhance the anti-adhesive and antibacterial performance of an MF membrane. For this reason, a PA membrane was chemically functionalized using silver (Ag) NPs and poly(carboxybetaine acrylate-*co*-dopamine methacryamide) (PCBDA). Fourier transform infrared (FTIR) and X-ray photoelectron spectroscopy (XPS) confirmed the formation of covalent bonds between catechol and amino groups. The surface hydrophilicity of the modified membrane was enhanced (WCA: ~30°) compared with that of the unmodified PA membrane (WCA: ~70°). The leaching test demonstrated that the amount of Ag lost after one week was approximately 3%, confirming the high stability of Ag within the PA/AgNP/PCBDA membrane structure. Moreover, the modified membrane showed the excellent ability to remove bacteria (99.6%) and coliform (100%) from river water in comparison with an unmodified PA membrane (33.34 and 62.07%, respectively). The unmodified PA membrane exhibited poor anti-adhesive properties by adsorbing 230 μg·cm^−2^ of bovine serum albumin (BSA) on its surface, while this value decreased to 12 μg·cm^−2^ for the modified membrane (after 1 day of immersion). Such results confirm the excellent anti-adhesive and antibacterial properties of a PA/AgNP/PCBDA membrane due to the chemical functionalization and enhanced hydrophilicity of a PA membrane [129].

Farjadian et al. [135] functionalized polypropylene (PP) MF membranes using a UV irradiation strategy to introduce poly(2-hydroxyethyl methacrylate) (PHEMA) chains onto the membrane surface. Then, the free amino groups in these chains reacted with aldehydes to form Schiff base functional groups, enabling the membranes to adsorb metal ions from water. Brunauer–Emmett–Teller (BET) analysis showed that the specific surface area of the porous PP membranes decreased from 27.2 (unmodified PP) to 22.1 m^2^·g^−1^ (functionalized membrane), confirming the reduction in the pore size of the MF membranes. These results were consistent with SEM findings, which indicated that the main reason was the coverage of the pores after the functionalization steps. It was observed that the modified membranes showed a lower water permeability (8600–11,100 L·m^−2^·h^−1^·bar^−1^) than the unmodified PP membrane (11,900 L·m^−2^·h^−1^·bar^−1^) owing to the decreased pore size during the modification steps. Moreover, the functionalized membrane showed a high adsorption capacity of ~90 mg·g^−1^ for copper ions, confirming the strong potential of the modified membranes for metal adsorption from water.

Another extensively studied chemical modification method for MF membranes is the grafting technique, where modified monomers and polymers are covalently bonded to the membrane surface (with a negligible change in the membrane bulk). This method is generally applied to address the limitations of MF membranes—such as poor selectivity, low hydrophilicity, and fouling phenomenon—by grafting a hydrophilic monomer or polymer onto the membrane surface. There are some unique positive properties of the chemical grafting method, including great stability, customizable surface properties, and broad material compatibility. Despite its advantages, this method presents few limitations, such as processing complexity, limited scalability, reduced permeability, and potential environmental impact. Wang et al. [130] prepared poly (sulfobetaine methacrylate) (PSBMA)-*graft (g)*-PVDF MF membranes via the chemical grafting strategy. The main aim of that research was to enhance the poor hydrophilicity of a PVDF membrane by grafting PSBMA at varying grafting degrees (GD) (2, 6, 10, and 14%). The bare PVDF membrane showed a WCA of ~130°, whereas PSBMA-*g*-PVDF membranes showed lower WCA values and by increasing GD, the WCA further decreased (from ~69 to 2°), confirming the enhancement of the hydrophilic properties of modified membranes. In addition, chemically modified membranes demonstrated higher porosity values (within the interval ~73–77%) in comparison with a bare PVDF membrane (~66%), verifying changes in membrane morphology. It was found that the modified membrane containing 10% GD showed the highest pure water flux of 105 L⸱m^−2^⸱h^−1^ compared with an unmodified PVDF membrane (60 L⸱m^−2^⸱h^−1^). It was observed that by increasing the GD from 10 to 14%, the pure water flux reduced owing to the pore clogging caused by PSBMA accumulation. Furthermore, PSBMA-*g*-PVDF membranes showed a higher FRR (up to 81%) than a bare PVDF membrane (65%) in BSA filtration. Such observations confirm the superior hydrophilic, transport, and antifouling properties of the grafted membrane compared with the bare PVDF membrane [130].

**Figure 20:**
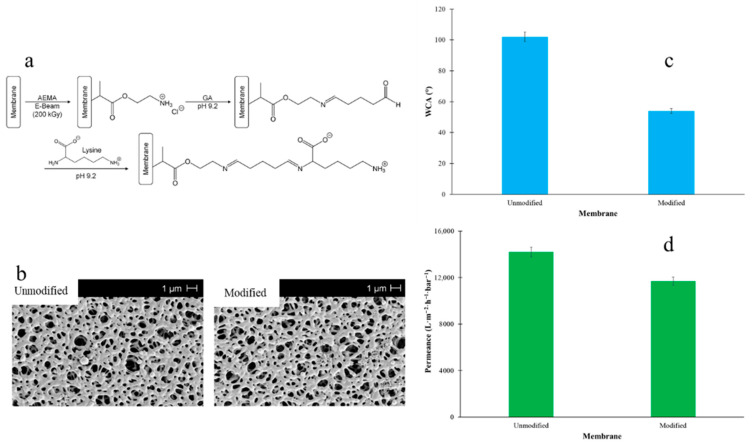
(**a**) Schematic of membrane modification; (**b**) SEM micrographs; (**c**) WCA; (**d**) performance of PES-based MF membranes (adapted from Breite et al., 2019) [136].

In another study, Breite et al. [136] modified PES MF membranes using a surface-initiated graft polymerization method. For this, a PES membrane was initially fabricated employing the NIPS method. Subsequently, the membrane surface was chemically modified via grafting zwitterionic polymer chains, introducing pH-responsive groups (Figure 20a). SEM images showed the same apparent pore size for both modified and unmodified membranes. This observation indicates that the modification steps did not significantly influence membrane morphology (Figure 20b). However, the modified membrane showed a lower WCA value of ~54° compared with the unmodified PES membrane (~102°), due to the presence of polar functional groups in the structure of the modified MF membrane (Figure 20c). Moreover, the modified membrane exhibited higher permeance than the pure PES membrane, owing to its enhanced hydrophilicity (Figure 20d). The findings indicate that the hydrophilic and transport properties of MF membranes can be enhanced by grafting polar functional groups.

Chemical grafting can be performed as a premodification technique to improve the physical, hydrophilic, transport, and antifouling properties of MF membranes. Ndlwana et al. [137] enhanced the hydrophilic, transport, and antifouling performance of PES MF membranes using a graft polymerization method as a premodification technique. They modified PES through free radical graft polymerization, in which methacrylic acid (MAA) was grafted onto PES in an aqueous medium using AIBN as a thermal initiator, forming PES-*g*-PMAA. Four MF membranes with different degrees of grafting (0 (M_0_), 2.6 (M_1_), 3.7 (M_2_), and 5.8% (M_3_)) were fabricated using the NIPS method. The unmodified membrane showed a WCA of 69°, while this value was considerably reduced for modified membranes (to between 53 and 42°). This observation can be explained by the enhanced hydrophilicity of grafted PES owing to the strong hydrophilic properties of PMAA. Moreover, the modified membranes exhibited a higher porosity (85–93%) than the pure PES membrane (51%), indicating an alteration in membrane morphology. The modified membranes showed a higher water flux (up to ~650 L·m^−2^·h^−1^) than the pure PES membrane (~325 L·m^−2^·h^−1^) because of the enhanced hydrophilicity and porosity of PES-*g*-PMAA membranes. Furthermore, the modified membranes showed excellent BSA rejection and flux recovery ratios of 97% and 86%, respectively, confirming the improved antifouling properties of modified membranes compared with the pure PES membrane.

**Figure 21:**
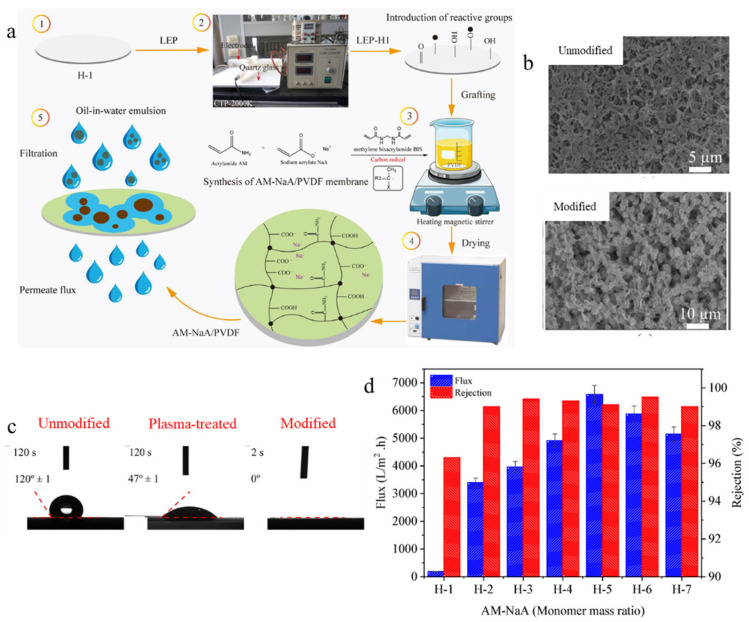
(**a**) Schematic of membrane modification; (**b**) SEM micrographs; (**c**) WCA; (**d**) flux and rejection performance of PVDF-based MF membranes (adapted from Hayder et al., 2025) [138].

In another study, Hayder et al. [138] modified PVDF MF membranes by grafting an acrylamide–sodium acrylate (AM-NaA) copolymer hydrogel onto its surface. Initially, the pure PVDF membrane was treated with low-temperature plasma to introduce functional groups. This step made the surface of the PVDF membrane more active, allowing the hydrophilic AM and NaA monomers to attach during subsequent graft polymerization (Figure 21a). The SEM images showed a uniform distribution of two hydrophilic monomers on the modified PVDF/AM-NaA membrane (Figure 21b). In addition, the hydrophilicity of the PVDF membrane was significantly enhanced in the plasma-treated and modified PVDF/AM-NaA membranes, owing to the presence of reactive polar functional groups and monomers on the surface of these membranes (Figure 21c). Moreover, PVDF/AM-NaA membranes exhibited higher flux and rejection performance (6579 L·m^−2^·h^−1^ and 99%, respectively) than the pure PVDF membrane, owing to the enhanced hydrophilic properties (Figure 21d). These findings indicate that the hydrophilic and transport properties of MF membranes can be enhanced by combining plasma treatment and chemical grafting strategies.

In addition to the mentioned methods, the in situ nanoparticle immobilization technique is a frequently used approach to chemically modify MF membranes. The term “in situ” is Latin for “on site”, indicating that the chemical modification (chemical reaction) occurs directly on the membrane surface or within its structure. This modification method involves three steps: 1. membrane surface preparation, 2. membrane surface activation, 3. in situ reaction. Generally, in the in situ nanoparticle immobilization technique, NPs are generated and anchored onto the membrane surface. Although, this method can enhance the hydrophilic and antibacterial properties of MF membranes, excessive NP loading may lead to pore blockage (due to NP accumulation), leading to flux reduction and potential NP leaching. However, the stability of NPs on/in the membrane surface/structure is much more than the physical incorporation method. Zhang et al. [131] modified a PVDF membrane using the in situ nanoparticle immobilization technique so as to improve the hydrophilic and transport properties of PVDF membrane. For this reason, a commercial MF membrane was initially immersed in tannic acid (TA) solution containing many phenolic hydroxyl groups (improving membrane hydrophilicity). Then, the PVDF/TA membrane was immersed in another silver nitrate (AgNO_3_) solution containing different concentrations (0.1, 0.2, 0.4, and 0.6 mg·mL^−1^) and for different immersing times (1, 2, 3, and 4 h). In this step, TA reduced the silver ions and AgNPs were chemically formed and anchored onto the membrane surface. A similar modification was completed with iron (II) sulfate (FeSO_4_) and copper (II) sulfate (CuSO_4_) solutions to prepare PVDF/TA/Fe and PVDF/TA/Cu membranes. The WCA was measured in dynamic mode for pristine and modified membranes, whereas no change in WCA was observed for a pristine PVDF membrane (~124°). The obtained results showed that the water droplet was absorbed by PVDF/TA/AgNP in approximately 10 s, while the absorption times for PVDF/TA/Fe and PVDF/TA/Cu membranes were ~17 and 33 s, confirming the better hydrophilicity of the PVDF/TA/AgNP membrane. Furthermore, the best hydrophilicity was reported for PVDF/TA/AgNP containing 0.4 mg·mL^−1^ Ag^+^ and 3 h immersion time. The PVDF/TA membrane demonstrated a higher pure water flux (5026 L⸱m^−2^⸱h^−1^) than the PVDF/TA/AgNPs membrane (4541 L⸱m^−2^⸱h^−1^) owing to the presence of numerous phenolic hydroxyl groups. Nonetheless, the PVDF/TA/AgNP membrane showed higher oil rejection and emulsion flux values (98.41% and 1431 L⸱m^−2^⸱h^−1^) compared with the PVDF/TA membrane. Such observations confirm that the poor hydrophilic properties of the PVDF membrane were significantly improved by the in situ nanoparticle immobilization modification method [131]. In another study, Zhang and colleagues [132] modified and developed a PVDF membrane using a multi-step chemical modification method. The main aim of that research was to enhance the omniphobic and antifouling properties of the PVDF MF membrane for application in the MD process. As a result, a PVDF porous membrane was initially coated with polydopamine (PDA) to introduce active functional groups (catechol and amine groups) onto the membrane. The next step was the in situ growth of TiO_2_ nanoparticles, which were immobilized on the PVDF/PDA membrane. The final modification step was carried out to reduce the surface free energy of the membrane through a fluorosilane grafting reaction (PVDF/PDA/F-TiO_2_ membrane). It was found that the PVDF/PDA/F-TiO_2_ membrane showed lower pore size of ~0.28 μm than the unmodified PVDF membrane (~0.35 μm). Additionally, the PVDF/PDA/F-TiO_2_ membrane revealed excellent omniphobic properties, with a high CA for pure water and mineral oil emulsion (~167° and 159°, respectively), which was significantly higher than the unmodified PVDF membrane (~127° and 68°, respectively). The PVDF membrane showed a great permeation flux of 37.5 kg⸱m^−2^⸱h^−1^ for multicomponent feed (containing NaCl, CaCl_2_, MgSO_4_, humic acid, and sodium dodecyl sulfate), while this value noticeably decreased over time, reaching 6.25 kg⸱m^−2^⸱h^−1^ after 24 h of operation. However, the PVDF/PDA/F-TiO_2_ membrane exhibited a stable permeation flux in the range of 34 to 36 kg·m^−2^·h^−1^, with no significant reduction over time. Such observations confirm the enhancement of the omniphobic and antifouling properties of the PVDF membrane through a multi-step chemical modification method [132]. Table 5 presents an overview of the merits and demerits of different chemical modification methods for MF membranes. Furthermore, Table 6 offers a summary regarding the various chemical modification strategies for polymeric MF membranes.

## 4. Strategic Vision for Future Research

**Figure 22:**
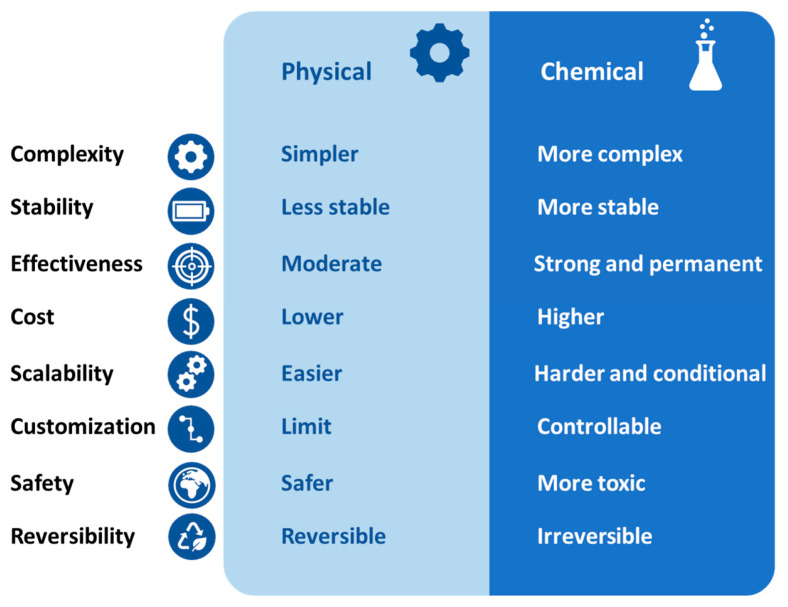
Comparison of physical and chemical modification methods for polymeric MF membranes.

Until now, numerous investigations have been conducted to fabricate and develop superior MF membranes with outstanding hydrophilic, transport, mechanical, antibacterial, and antifouling properties while also emphasizing environmental sustainability and economic viability [108,109,110,111,112]. Nevertheless, the identification of MF membranes possessing such exceptional characteristics remains a subject of ongoing discussion and scrutiny within the scientific community. We are currently encountering the question: what is the optimal modification method for polymeric MF membranes—physical or chemical? The findings indicated that physical and chemical modification methods present distinct advantages and limitations (Figure 22) [113,114,115,127,128,129]. On the one hand, physical modification methods are simple, cost-effective, scalable, and environmentally friendly, while this type of modification technique cannot offer MF membranes with stable transport and antifouling properties owing to the lack of chemical interactions [31,116,117,118]. On the other hand, chemical modification techniques take advantage of strong chemical interactions—such as covalent bonding—to prepare MF membranes with stable transport, antibacterial, and antifouling properties [111,112,127,130]. However, chemical modification methods are usually complex, time-intensive, and expensive with a higher risk of environmental pollution (compared with the physical methods) due to the use of extra toxic reagents [129,130,131,132].

Considering these trade-offs, benefiting from a hybrid modification method may be a promising approach to obtain a MF membrane possessing great hydrophilic, morphological, transport, and antifouling properties. Membranes modified with a hybrid approach can take advantage of the simplicity, scalability, and cost-effectiveness of physical methods, and they can benefit from the durability and performance stability supplied by chemical modification techniques. The individual limitations of each method can be overcome by using this strategy. For instance, physical modifications (e.g., amphilic copolymer integrating or NP incorporation) can enhance the hydrophilic, transport, and antibacterial properties of the membrane, whereas chemical ones (e.g., chemical crosslinking or grafting) can be employed for the stabilization of these modified properties for long-term applications. Taking advantage of this synergistic integration makes it possible to prepare a new generation of MF membranes with superior permeate flux and antifouling properties. Furthermore, this strategy offers excellent flexibility in engineering and developing membrane features for specific applications, making them promising candidates for both laboratory and industrial scales.

Moreover, in future research, more focus should be given to application-specific requirements when choosing and developing modification methods for MF membranes. Each scientific and industrial sector has unique performance requirements. For instance, on the one hand, the pharmaceutical and biomedical industries prioritize membrane biocompatibility and disinfectability. On the other hand, wastewater treatment and industrial effluent management focus on the engineering and development of MF membranes with long-term antifouling capabilities under harsh operating conditions. As a result, the ideal modification method cannot be certainly defined and must be adapted to the intended application. Future investigations should aim to establish structured frameworks that link end-use demands with corresponding physical, chemical, or hybrid modification techniques. Therefore, this approach enables rational membrane design and drives the development of next-generation MF membranes optimized for specific applications.

Another interesting way to develop MF membranes is the integration of artificial intelligence (AI) and machine learning (ML) into MF technology. This recent field of science can be employed to evaluate large experimental datasets to identify correlations between membrane composition, fabrication parameters, and performance metrics. For example, ML algorithms can facilitate the adjustment of polymer blend compositions, NP loading level, and operating parameters to enhance permeability while minimizing fouling. In addition, predictive models powered by AI can be employed to estimate membrane service life, fouling tendencies, and cleaning efficiency. Consequently, this technology saves time and energy by reducing experimental trial-and-error and accelerating membrane design. Moreover, AI could assist in identifying novel materials (i.e., polymers and NPs) and modification methods by screening and analyzing extensive databases of potential materials, resulting in the identification of superior materials (i.e., high-performance candidates) that might not be discovered through traditional and conventional experimental approaches alone.

Despite the fact that significant academic research exists, the number of modification techniques that have been successfully implemented at the industrial scale remains limited. To date, commercially applied modification strategies are mostly restricted to polymer blending (with porogens) and surface coating, as these methods are scalable, efficient, and cost-effective. Even though advanced chemical modification and functionalization methods are highly effective at the laboratory scale, high cost, environmental safety considerations, and limited scalability are the main drawbacks of these methods. Overcoming this gap will require future investigations to address not only on membrane performance, but also process scalability, life-cycle assessment, and adherence to industrial and environmental regulations.

## 5. Conclusions

For decades, polymeric microfiltration membranes have been widely employed in many separation processes—including water treatment and various food and pharmaceutical applications—thanks to their excellent flux permeance, moderate selectivity, and cost-effectiveness. Despite these merits, polymeric microfiltration membranes still suffer from fouling phenomenon, causing reduced flux, shortened service life, and increased energy demand. Consequently, extensive research has been devoted to tailoring efficient strategies to limit fouling and boost membrane performance.

Even though numerous studies have individually examined physical and chemical modification strategies, this review uniquely provides a systematic and comparative assessment of both modification techniques, such as polymer blending, the incorporation of nanomaterials, surface coating, chemical crosslinking, in situ nanoparticle immobilization, and chemical surface functionalization. By critically examining these techniques side by side, this review not only consolidates scattered findings in the literature but also discusses the advantages and limitations of each method, along with key findings from recent research.

Furthermore, this review provides integrated insights into how different modification methods influence the physical, hydrophilic, morphological, transport, and antifouling properties of polymeric microfiltration membranes, giving a clearer overall picture than previous reports. Additionally, by highlighting the key knowledge gaps and persistent challenges of each modification method, this review suggests forward-looking insights and promising approaches for next-generation polymeric microfiltration membranes.

Overall, this review enhances the current body of literature by offering a framework for scientists and engineers to facilitate the selection and design of modification techniques to obtain polymeric microfiltration membranes with improved efficiency, extended service life, and great mechanical stability. The insights provided in this study are expected to guide future studies and accelerate the development of high-performance polymeric microfiltration membranes which can truly meet the demands of industrial applications.

## Figures and Tables

**Table 1:** Summary of studies reporting quantitative flux decline caused by fouling in polymeric MF membranes.

Membrane	Pore Size (μm)	Feed/Solution Filtered	WCA Range (°)	Flux Decline (%)	Ref.
PVDF PES	0.22, 0.45	Mulberry wine	PVDF: 64–82 PES: 44–53	PVDF: 55–66 PES: 65–66	[104]
PVDF PVDF-*g*-P(HEMA-*co*-NIPAAm)	0.45	Bacterial suspensions	–	Unmodified: 63–83 Modified: 8–70	[105]
PE PE/PMEA	0.06	Sea water	–	Unmodified: 34 Modified: 19	[106]
PES	0.22	HA–BSA	28–65	40–85	[107]
PVDF	0.1	Synthetic cyanide solution	–	50–55	[57]

**Table 2:** Key factors influencing MF fouling and optimal conditions for weaker fouling.

Category	Factor	Optimal Condition
Feed characteristics	Particle size	Larger than membrane pore size
	Organic content	Low concentration
	pH	Far from IEP
Operating condition	Flux	Below critical flux
	Transmembrane pressure	Low
	Filtration type	Cross-flow filtration
	Temperature	Moderate
Membrane properties	Pore size	Not similar to foulant size
	Surface roughness	Low (i.e., smooth surface)
	Hydrophilicity	High

**Table 3:** Summary of the advantages and drawbacks of various physical modification techniques applied to polymeric MF membranes.

Physical Method	Description	Advantages	Disadvantages
Polymer blending	Blending hydrophilic polymers (e.g., PVA, chitosan) with base polymer (e.g., PS, PVDF)	▪ Improves hydrophilicity ▪ Increases water flux ▪ Reduces fouling	▪ Possible phase separation ▪ Leaching of additives over time ▪ Limited long-term stability
Nanoparticle incorporation	Incorporation of NPs (e.g., TiO_2_, SiO_2_, ZnO, GO) into the polymer matrix	▪ Improves hydrophilicity and permeability ▪ Enhances antifouling ▪ Potential antimicrobial properties	▪ Nanoparticle aggregation ▪ Uneven dispersion affects performance ▪ Cost and long-term stability concerns
Amphilic copolymer integration	Incorporating copolymers with hydrophilic and hydrophobic blocks	▪ Hydrophilic blocks improve surface wettability ▪ Strong anchoring improves stability	▪ More difficult and costly synthesis ▪ Optimization required for compatibility
Surface coating	Applying a thin hydrophilic or functional layer onto the membrane surface	▪ Enhances hydrophilicity ▪ Improves water flux ▪ Enhances fouling resistance	▪ Coating may peel or degrade ▪ Adds extra fabrication step
Plasma treatment	Exposing membrane surface to reactive plasma (O_2_, N_2_, air, and NPs)	▪ Introduces polar functional groups ▪ Increases surface energy ▪ Increases adhesion for coatings	▪ Surface effects may be temporary (hydrophobic recovery) ▪ Limited penetration into pores
Thermal pressing/annealing	Controlled heating post-fabrication to modify polymer chains	▪ Changes pore structure and porosity ▪ Enhances mechanical strength	▪ Excessive heat may collapse pores ▪ Minimal improvement in hydrophilicity
Physical parameter optimization	Adjusting preparation conditions (polymer conc., solvent, coagulation bath, temperature)	▪ Controls pore size and distribution ▪ Optimizes water flux and selectivity ▪ No chemical use required	▪ Process-dependent, trial and error ▪ Limited ability to introduce new functionality

**Table 4:** Summary of MF membranes modified by physical modification strategies.

Modification Method	Membrane Composition	Polymer Base	Highest Pure Water Flux (L⸱m^−2^⸱h^−1^)/Permeance (L⸱m^−2^⸱h^−1^·bar^−1^)	WCA Range (°)	Highest Rejection Rate (%)	Ref.
Polymer blending	PS/PEI	PS	342.17 ǀ –	Before modification: 86 After modification: 68–78	Sulfate: 99.6 Chloride: 92.3 Iron: 94.6 Manganese: 97.5 TOC: 79.1 Turbidity: 98.3	[114]
	PVDF/PVA	PVDF	~1600 ǀ –	Before: 87 After: 57–64	–	[119]
	PS/PEO	PS	97 ǀ –	–	Phenol: 60	[120]
Nanoparticle incorporation	SAN/AgNPs	SAN	511 ǀ –	Before: 18 After: 15–76	E-coli: 94.0 S-aureus: 98.0	[113]
	BPPO/SiO_2_	BPPO	30 × 10^3^ ǀ –	Before: 105 After: 47–69	–	[121]
	PS/PEI/TiO_2_ PS/PEI/Al_2_O_3_	PS	2800 ǀ –	Before: 94 After: 69–86	Turbidity: 99.9	[122]
Amphilic copolymer integration	PES/P(MAPEG-*co*-MALA-*co*-MAHA)	PES	89 ǀ –	Before: 74 After: 54–62	BSA: 77.3 Au NPs: 66.2	[115]
	PVDF/P(S-*r*-EGMA)	PVDF	9500 ǀ –	Before: 131 After: 94–126	Bacteria: 97.0	[123]
Surface coating	PVDF/TP-silica	PVDF	15,353 ǀ –	Before: 109 After: 24–52	Emulsion: 97.0	[116]
	PA/TiO_2_-AgO	PA	~4700 ǀ –	–	E-coli: 99.9 B-subtilis: 99.9 MB: 68-99	[91]
Plasma treatment	PES	PES	16,000 ǀ –	Before: 60 After: 10–20	Microbe: 92.4	[117]
	PA/Zn PA/ZnO PA/Zn-ZnO	PA	~4100 ǀ –	Before: 98 After: 84–105	E-coli: 99.9 S-aureus: 99.9	[124]
	PA/AgO	PA	~4900 ǀ –	–	E-coli: 99.9 B-subtilis: 99.9	[125]
Thermal pressing/annealing	PVDF-*co*-HFP	PVDF-*co*-HFP	35 (for NaCl 3.5wt.%) ǀ –	Before: 153 After: 142–147	NaCl: 99.9	[118]
	PS	PS	834 ǀ –	115–137	–	[66]
Physical parameter optimization	PA6	PA6	28.6 ǀ –	–	PEG: 50	[31]
	PI	PI	– ǀ 2.91 (for 2-PrOH-based solution)	–	RB: 99.7	[126]

**Table 5:** Overview of the advantages and disadvantages associated with various chemical modification techniques for polymeric MF membranes.

Chemical Method	Description	Advantages	Disadvantages
Crosslinking	Formation of covalent bonds to enhance structural stability	▪ Increases chemical, thermal, and mechanical stability ▪ Enhances fouling resistance	▪ Can reduce membrane permeability if over-crosslinked ▪ Often requires additional chemicals and processing steps
Chemical Surface Functionalization	Introducing functional groups (–OH, –COOH, –NH_2_, etc.) via chemical reactions on the membrane surface	▪ Improves hydrophilicity and surface wettability ▪ Enhances antifouling properties ▪ Tailorable surface chemistry	▪ Can involve harsh chemicals ▪ Surface modification may affect bulk properties if not controlled ▪ More complex process
Chemical Grafting	Covalent attachment of polymers or molecules onto membrane surface	▪ Stable, long-lasting surface modification ▪ Introduces specific functionalities ▪ Improves fouling resistance	▪ Requires specialized chemistry ▪ Time-consuming and potentially costly
In Situ Nanoparticle Immobilization	Formation or deposition of NPs directly onto or within the membrane during chemical reaction	▪ Strong nanoparticle adhesion ▪ Enhances hydrophilicity, permeability, and antifouling ▪ Provides antimicrobial properties	▪ Process complexity is higher ▪ NP aggregation or uneven distribution possible ▪ Additional chemical reagents required

**Table 6:** Summary of polymeric MF membranes modified by chemical modification strategies.

Modification Method	Membrane Composition	Polymer Base	Highest Pure Water Flux (L⸱m^−2^⸱h^−1^)/Permeance (L⸱m^−2^⸱h^−1^·bar^−1^)	WCA Range (°)	Highest Rejection Rate (%)	Ref.
Crosslinking	PCTE/BDG/BPEI	PCTE	– | 1050	Before modification: 61 After modification: 68	Glass sphere: 100	[127]
	PTFE/PEGML/PVA/CA	PTFE	397 | –	Before: 82 After: 38	–	[133]
	PP/GA/Sericin	PP	7.97 | –	Before: 82 After: 38	Turbidity: >90	[128]
	PVDF/PVA	PVDF	– | 5500	Before: 101 After: 45	Oil: 98	[134]
Chemical functionalization	PA/AgNP/PCBDA	PA	– | –	Before: 70 After: 30	Bacteria: 99.6 Coliform: 100	[129]
	PP/PHEMA	PP	– | 11,900	–	–	[135]
Chemical grafting	PVDF-*g*-PSBMA	PVDF	105 | –	Before: 130 After: 2–69	–	[130]
	PES/AEMA/lesine	PES	– | 14,200	Before: 102 After: 54	–	[136]
	PES PES-*g*-PMAA	PES	650 | –	Before: 69 After: 42–53	BSA: 97	[137]
	PVDF/AM-NaA	PVDF	6579 (for oil/water) | –	Before: 120 After: 0–47	Oil: 99	[138]
In situ nanoparticle immobilization	PVDF/TA/AgNP PVDF/TA/Fe PVDF/TA/Cu	PVDF	4541 | –	Before: 124 After: 21–31	Oil rejection: 98.4	[131]
	PVDF/PDA PVDF/PDA/TiO_2_ PVDF/PDA/F-TiO_2_	PVDF	36 kg⸱m^−2^⸱h^−1^ (for multicomponent feed solution) | –	Before: 127 After:167	Salt rejection: 100	[132]

## Data Availability

No new data were created or analyzed in this study.

## Abbreviations

2-PrOH	2-Propanol
AgNO_3_	Silver nitrate
AgNPs	Silver nanoparticles
AI	Artificial intelligence
Al_2_O_3_	Alumina (aluminum oxide)
BDG	1,4-butanediol diglycidyl ether
BET	Brunauer–Emmett–Teller analysis
BPEI	Branched polyethylenimine
BPPO	Brominated polyphenylene oxide
BSA	Bovine serum albumin
CNPs	Carbon nanoparticles
CuSO_4_	Copper (II) sulfate
FeSO_4_	Iron (II) sulfate
FRR	Flux recovery ratio
FTIR	Fourier transform infrared spectroscopy
g	Graft
GA	Glutaraldehyde
GD	Grafting degree
GO	Graphene oxide
HA	Humic acid
HPP	Hierarchical porous polymer
IEP	Isoelectric point
MAHA	4-formylphenyl-2-methylprop-2-enoate
MALA	2-(methacryloyloxy)ethyl 5-(1,2-dithiolan-3-yl)pentanoate
MAPEG	(ethyleneglycol)methylethermethacrylate
MD	Membrane distillation
MF	Microfiltration
ML	Machine learning
NF	Nanofiltration
NIPS	Non-solvent induced phase inversion
NTIPS	Non-solvent thermally induced phase inversion
NP	Nanoparticle
PA	Polyamide
PA6	Polyamide-6
PCBDA	Poly(carboxybetaine acrylate-*co*-dopamine methacryamide)
PCTE	Polycarbonate track-etched
PE	Polyethylene
PEG	Polyethylene glycol
PEGML	Polyethylene glycol laurate
PEI	Polyetherimide
PEO	Polyethylene oxide
PES	Polyethersulfone
P(HEMA-*co*-NIPAAm)	Poly(hydroxyethyl methacrylate)-*co*-(*N*-isopropylacrylamide)
PI	Polyimide
PMEA	Poly(2-methoxyethyl acrylate)
PP	Polypropylene
PS	Polysulfone
PSBMA	Poly (sulfobetaine methacrylate)
P(S-*r*-EGMA)	Poly(styrene-*r*-(ethylene glycol) methyl ether methacrylate)
PTFE	Polytetrafluoroethylene
PVDF	Polyvinylidene fluoride
PVP	Polyvinylpyrrolidone
RB	Rose Bengal
RO	Reverse osmosis
SAN	Poly(acrylonitrile-styrene)
SEM	Scanning electron microscopy
SiO_2_	Silica
TA	Tannic acid
TiO_2_	Titanium dioxide
TPs	Tea polyphenols
TOC	Total organic carbon
UF	Ultrafiltration
VIPS	Vapor-induced phase inversion
WCA	Water contact angle
XPS	X-ray photoelectron spectroscopy
ZnO	Zinc oxide
ZrO_2_	Zirconia
